# Enhancing Tendon Regeneration: Investigating the Impact of Topography on the Secretome of Adipose‐Derived Stem Cells

**DOI:** 10.1002/advs.202417447

**Published:** 2025-03-17

**Authors:** Qiuzi Long, Chuanquan Liu, Haotian Zheng, Mingyue Wang, Hanmei Liu, Yue Liu, Zhicheng Cao, Yuzhi Sun, Qingyun Mo, Ludvig J. Backman, Jialin Zhu, Lizhi Hu, Jinlong Huang, Wei Zhang, Jialin Chen

**Affiliations:** ^1^ Nanjing University of Chinese Medicine Nanjing 210029 China; ^2^ Center for Stem Cell and Regenerative Medicine Southeast University Nanjing 210009 China; ^3^ Nanjing Second Hospital Nanjing Hospital affiliated to Nanjing University of Chinese Medicine Nanjing 210003 China; ^4^ School of Medicine Southeast University Nanjing 210009 China; ^5^ Department of Orthopaedic Surgery Institute of Digital Medicine Nanjing First Hospital Nanjing Medical University Nanjing 210006 China; ^6^ Department of Medical and Translational Biology, Anatomy Umeå University Umeå 90187 Sweden; ^7^ Department of Community Medicine and Rehabilitation Umeå University Umeå 90187 Sweden; ^8^ Jiangsu Key Laboratory for Biomaterials and Devices Southeast University Nanjing 210096 China; ^9^ China Orthopedic Regenerative Medicine Group (CORMed) Hangzhou 310058 China; ^10^ Department of Ophthalmology Zhongda Hospital Southeast University Nanjing 210009 China

**Keywords:** ADSCs, paracrine, proteomics, tendon regeneration, topology

## Abstract

Tendons are vital for maintaining integrity and movement, but current treatment options are insufficient for their regeneration after injuries. Previous studies have shown that the secretome from mesenchymal stem cells (MSCs) promoted tendon regeneration. However, limited studies have explored the impact of the physical microenvironment on the secretome's efficacy of MSCs. In this study, it is shown that the topographic orientation regulates the secretome of human adipose‐derived stem cells (ADSCs) and promotes tendon regeneration. Conditioned medium (CM) is collected from ADSCs cultured on the scaffolds with different topography. The results show that CM generated from aligned structure group has a potent effect in promoting cell migration and proliferation, tenogenic differentiation, macrophage polarization toward M2 phenotype, tendon structure and mechanical function recovery. Proteomic analysis revealed that the aligned structure can up‐regulate the secretion of Extracellular matrix (ECM) proteins while down‐regulate proinflammatory factors. This modulation activates the MAPK, GPCR and Integrin signaling pathways which may account for the enhanced effect on tendon regeneration. This study offers a promising and safer non‐cell‐based treatment option for tendon repair.

## Graphical abstract

The impact of topography on the secretome of adipose‐derived stem cells (ADSCs) has the potential for promoting tendon regeneration. The secretome generated from ADSCs on aligned structure has a potent effect in promoting cell migration, proliferation, tenogenic differentiation, macrophage M2 polarization, and tendon mechanical function recovery. Aligned structure can up‐regulate the ADSCs’ secretion of Extracellular matrix (ECM) proteins but down‐regulate proinflammatory proteins, which may account for the enhanced effect on tendon regeneration.

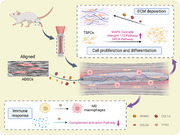

## 1 Introduction

Tendons play a crucial role in joint movement by attaching muscles to bones. Tendon injuries are a frequent occurrence in both athletic and non‐athletic populations and are a significant cause of musculoskeletal pain and can impact daily activities, leading to decreased quality of life for those affected.^[^ ^1^, ^2^ ^]^ Current treatment options for tendon injuries include conservative treatment (steroid injections, low‐intensity pulsed ultrasound, shockwave, and physical therapy) and surgeries.^[^ ^3^ ^]^ However, despite treatment, the gross, histological, or mechanical characteristics of the injured tendon are often not fully restored due to the formation of fibrovascular scars.^[^ ^4^, ^5^ ^]^ Numerous biomaterials and scaffolds have been studied for tendon repair, such as collagen sponge,^[^ ^6^ ^]^ silk‐based matrix,^[^ ^7^ ^]^ polypropylene‐based devices,^[^ ^8^ ^]^ PLGA‐based scaffolds,^[^ ^9^ ^]^ and electro‐spun collagen scaffolds.^[^ ^10^ ^]^ Despite the huge progress that has been achieved, it is yet to meet the requirements for functional tendon regeneration due to the the insufficient bioactivity of biomaterials/scaffolds.^[^ ^11^ ^]^ To enhance the bioactivity of scaffolds, exogenous biological factors were usually incorporated into the scaffolds.^[^ ^10^, ^11^, ^12^ ^]^ Instead, seed cells such as mesenchymal stem cells (MSCs) had good bioactivity, and therefore MSCs‐based scaffold‐free tendon tissue engineering has been a potential alternative for the treatment of tendon injury, offering new possibilities to overcome the limitations of current therapies.^[^ ^13^, ^14^ ^]^

The main MSCs used in tendon tissue engineering include bone marrow mesenchymal stem cells (BMSCs), adipose‐derived stem cells (ADSCs), tendon stem/progenitor cells (TSPCs), and umbilical cord mesenchymal stem cells (UCMSCs). As compared to other types of MSCs, ADSCs are considered advantageous due to their ease of accessibility and low cost.^[^ ^15^ ^]^ To some extent, ADSCs have shown significant superiority over BMSCs in repairing damaged tendons, as evident by their ability to restore tendon toughness, energy absorption capacity, maximum stress, and to promote cell proliferation.^[^ ^4^ ^]^ In addition, ADSCs can be harvested through a less invasive method, and have fewer ethical challenges as compared to other types of stem cells.^[^ ^16^ ^]^ Recent research suggests that the regenerative benefits of MSCs are mainly due to their secretome and its paracrine effects rather than their direct differentiation abilities.^[^ ^17^, ^18^ ^]^ The cytokines from MSCs coordinate various biological processes that promote tissue regeneration, including angiogenesis, cell recruitment, and differentiation.^[^ ^19^, ^20^ ^]^ It is shown that MSC exosomes play a significant role in tissue regeneration by exerting immunomodulatory effects. For example, MSC exosomes can promote M2 polarization of macrophages, and regulate the differentiation of Treg and Th2 cells, thus enhancing their pro‐regenerative capacity.^[^ ^21^, ^22^, ^23^, ^24^ ^]^ Secretome from other MSCs has been found to promote vitality of tendon cells in vitro and enhance tendon repair in vivo.^[^ ^25^ ^]^ ADSCs‐derived secretome has high levels of insulin‐like growth factor‐1 (IGF‐1), vascular endothelial growth factor‐D (VEGF‐D) and interleukin‐8 (IL‐8),^[^ ^26^ ^]^ making it promising for tendon repair. It was found that ADSCs exosomes facilitated the tendon healing by up‐regulating the expression of TNMD, TNC, and SCX in tendon stem cells (TSCs)^[^ ^27^ ^]^ and promoted the proliferation and migration of TSCs by both activating the SMAD2/3 and SMAD1/5/9 pathway.^[^ ^28^ ^]^ Besides, ADSCs conditioned medium (CM) showed the potential to treat diabetic tendinopathy by restoring the natural morphology, cell viability, and structure of tenocytes and promoting their scratch wound closure in a high glucose culture condition.^[^ ^29^ ^]^ Therefore, a potential strategy for tendon regeneration is to utilize the paracrine effect of ADSCs as a cell‐free treatment option.

The local microenvironment plays a critical role in influencing phenotype and secretory capacity of cells.^[^ ^26^ ^]^ The topography of the extracellular matrix (ECM) is a crucial element of the local microenvironment, affecting cell behaviors such as morphology, migration, proliferation, and differentiation.^[^ ^30^, ^31^, ^32^ ^]^ The aligned topology of the tendon is characterized by collagen fibers arranged sequentially in the direction of stress.^[^ ^33^ ^]^ Compared with random scaffolds, previous studies have demonstrated that aligned scaffolds can promote the directional arrangement of cells and deposited collagen fibers, as well as promote tendon‐lineage differentiation of TSPCs or other MSCs to achieve the effect of tendon regeneration.^[^ ^34^, ^35^, ^36^ ^]^ However, few studies have attempted to investigate whether topological structure can exert the role of physical regulation on cell paracrine to achieve the purpose of tendon repair. Aligned scaffolds can not only affect cell phenotype and arrangement, but also promote tissue repair by altering the secretory pattern and, consequently, the paracrine effect. Study have reported that the CM produced by MSCs cultured on aligned fiber sheets can more effectively promote MSCs proliferation compared to those on random fiber sheets.^[^ ^37^ ^]^ In addition, study have also shown that aligned microfibers enhance the paracrine function of macrophages, thereby promoting the maturation of Schwann cells for neural repair.^[^ ^38^ ^]^ Therefore, it is worth to explore the effect and mechanism of topological structure on the paracrine of stem cells and its potential for tendon repair and regeneration.

**Figure 1:**
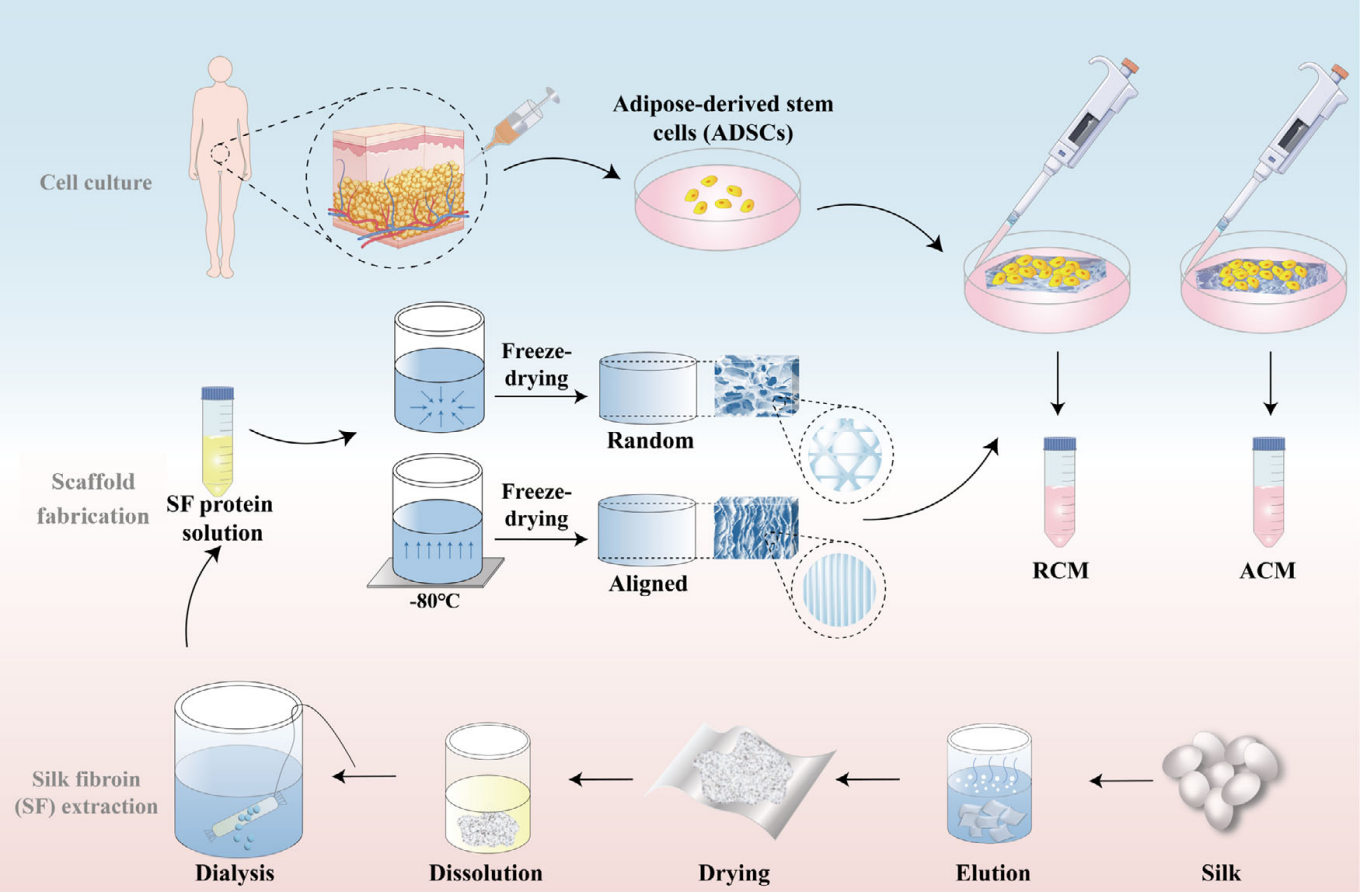
Preparation of aligned and random silk fibroin scaffolds and collection of ADSCs‐derived CM. CM, conditioned medium; RCM, ADSCs derived CM from random oriented silk fibroin scaffolds; ACM, ADSCs derived CM from aligned oriented silk fibroin scaffolds.

In this study, we prepared random and aligned silk fibroin (SF) scaffolds by using “directional freeze‐drying” (**Figure** **1**). Scaffolds with random and aligned topological orientation were used to culture ADSCs, and their CM generated from randomly oriented (RCM) and aligned oriented (ACM) SF scaffolds were collected. The effects of RCM and ACM on cell migration, proliferation, and tendinous differentiation of TSPCs, as well as its effect on macrophage polarization were compared to evaluate the potential regulatory effects of topological orientation on the secretome and paracrine function of ADSCs. Subsequently, freeze‐dried CMs were combined with SF‐based light‐triggered hydrogel and administrated into the defect of a rat patellar tendon injury model. After surgery, histological evaluation and examination of inflammatory markers were performed and the mechanical strength were evaluated by gait analysis and mechanical test. Finally, proteomic analysis of CMs was performed to analyze the effect of topography on paracrine proteins of ADSCs. The involvement of MAPK, GPCR, and Integrin1/3 pathways during the process was further investigated. Our data suggested that aligned topography induces a secretome with more proper paracrine responses from ADSCs, and potential for tendon regeneration.

## 2 Results

### 2.1 Characterization of ADSCs and Fabrication of Scaffolds with Different Topographies

**Figure 2:**
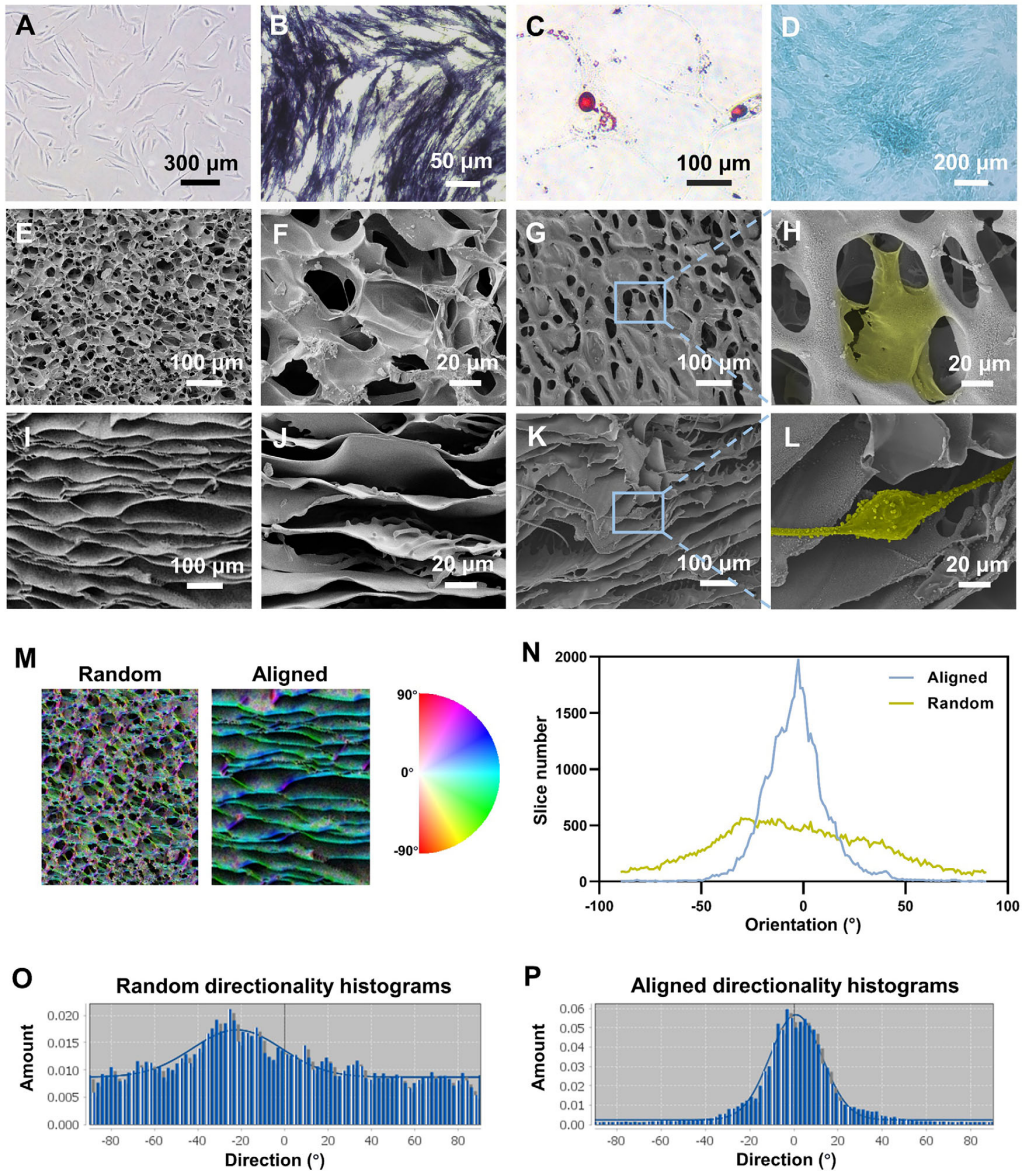
Characterization of ADSCs and scaffolds with different topographies. A) Cell morphology of ADSCs. B–D) Multi‐lineage differentiation properties of ADSCs were evaluated. ADSCs were cultured in osteogenic differentiation media for 1 week and evaluated by ALP staining (B). ADSCs were cultured in adipogenic differentiation media for 2 weeks and evaluated by staining lipid droplets with oil red O (C). ADSCs were cultured in chondrogenic differentiation media for 2 weeks and evaluated by Alician Blue staining (D). E–L) The topographic orientation of silk fibrin scaffolds and ADSCs cultured on them were visualized under SEM. E–H) Random scaffold. I–L) Aligned scaffold. M) Overlapping pore orientation color map showing degree of anisotropy in random and aligned scaffolds. N–P) Representative histogram curve in random and aligned scaffolds of the orientation shown in the color map images where the plugin from Fiji, Directionality, was used to calculate the number of fibers (Fiji count) per direction (degree).

The cultured ADSCs exhibited an elongated fibroblast‐like morphology under microscopy (**Figure** **2**A). These cells were found to be positive for CD73, CD90, and CD105, but did not express the markers CD34, CD11b, CD19, or CD45 by flow cytometry (Figure S1, Supporting Information). The multi‐lineage differentiation potential of ADSCs was assessed by culturing them in three distinct types of differentiation media. After one week of induction, the osteogenic differentiation potential of ADSCs was confirmed by ALP staining (Figure 2B). The adipose‐ and chondro‐lineage differentiation potential of ADSCs were confirmed by oil red O and Alician Blue staining respectively after two weeks of induction (Figure 2C,D).

Random and aligned silk scaffolds were fabricated and the pore structure was visualized under SEM (Figure 2E–L). The pores in the aligned group were distinct from those in the random group as they were elongated and aligned parallel to the direction of the temperature gradient, which was a result of the directional freezing process. The “OrientationJ” and “directionality” plugins for Fiji were used to quantify the degree of pore anisotropy (Figure 2M–P). The pore arrangement of the aligned structure scaffolds was mainly distributed in the range of −10° to 10°, while the pore arrangement in random structure scaffolds has a distribution in all direction (Figure 2N–P). The differences of cell morphology cultured on the scaffolds of the two topologies was visualized under SEM (Figure 2G,H,K,L). The morphology of the cells on the random scaffold was restricted by the pores, and the cell pseudopods were tightly bound to the surface of the scaffold. On the aligned scaffold, the cells were elongated with the direction of pores, and the cells were loosely bound to the scaffold.

### 2.2 Effects of RCM and ACM on TSPCs Migration and Cell Proliferation

**Figure 3:**
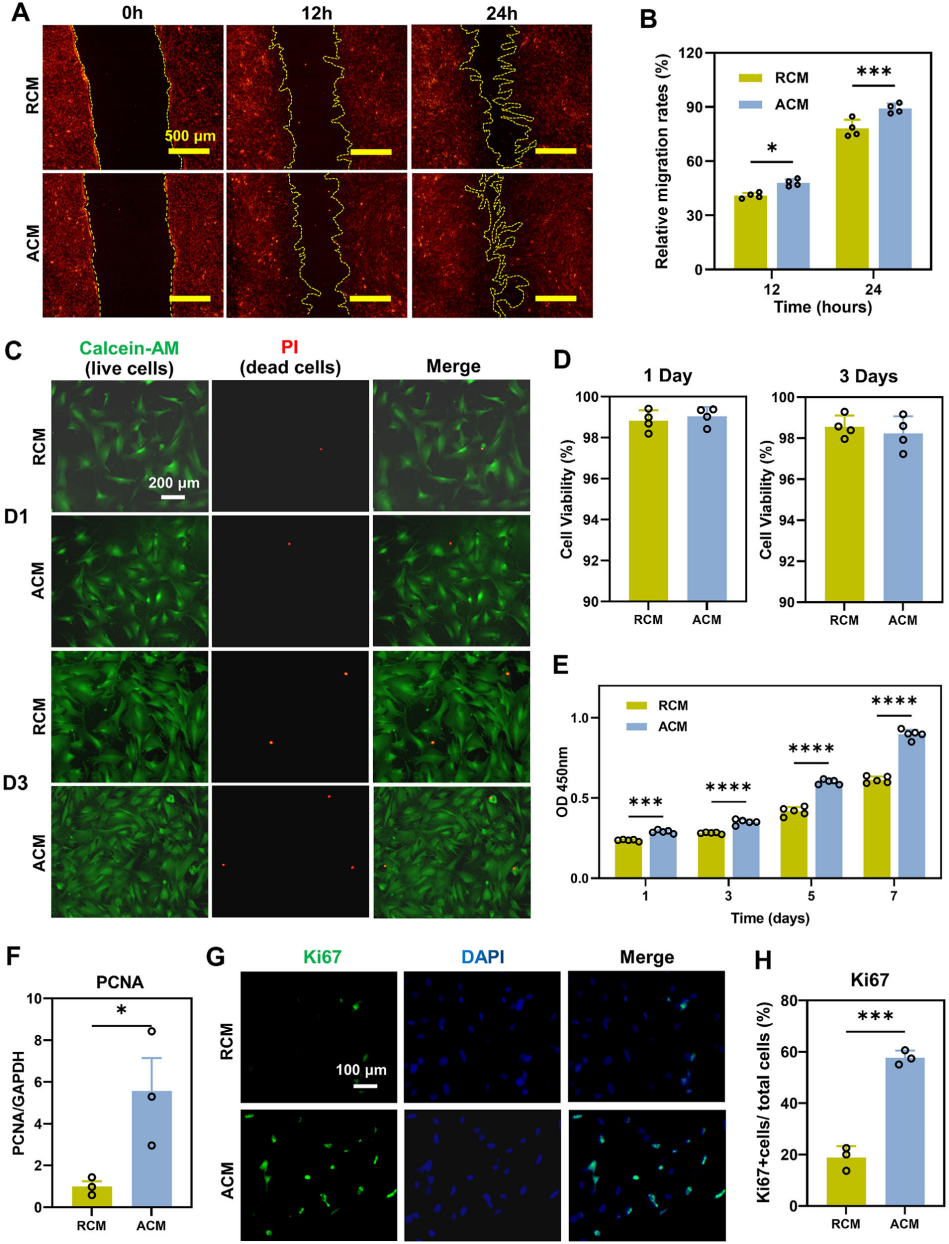
Effects of RCM and ACM on TSPCs migration and cell proliferation. A) Cell migration of TSPCs treated with RCM or ACM using scratch assay at 0, 12, and 24 h. TSPCs were pre‐stained using Dil. Scale bars = 500 µm. B) The relative migration rate was quantified and compared, *n* = 4 technically independent samples for each group, *p < 0.05, ***p < 0.001. C,D) Live/dead staining of TSPCs after 1 day and 3 days of culture in RCM and ACM (live cells in green and dead cells in red). Scale bar = 200 µm. The cell viability was calculated by ImageJ, *n* = 4 randomly‐selected microscopic images per group. E) Proliferation of TSPCs in RCM and ACM for 1, 3, 5, and 7 days measured by CCK‐8, *n* = 5 technically independent samples for each group, ***p < 0.001 ****p < 0.0001. F) Gene expression of PCNA in RCM and ACM at day 1. The level at the RCM group was set as 1. Data were shown as Mean ± SEM, *n* = 3 technically independent samples for each group, *p < 0.05. G) The expression of Ki67 was evaluated by immunofluorescence staining. Scale bar = 100 µm. H) Ki67+ cells/total cells were quantified and compared, *n* = 3 randomly‐selected microscopic images per group, ***p < 0.001.

ADSCs were seeded on randomly oriented or aligned silk scaffolds. RCM and ACM were collected after 24 h. To evaluate the effects of the ADSCs secretome on TSPCs, RCM and ACM were introduced to the cell culture of TSPCs on tissue culture plastic (TCP). Cell scratch assay was used to investigate the migration of TSPCs after RCM and ACM treatments. The results indicated that the migratory capacity of TSPCs was significantly higher in the ACM group, as compared to the RCM group (**Figure** **3**A,B). In addition, TSPCs in the RCM and ACM groups exhibited better cell migration ability compared to TSPCs cultured in medium collected from randomly oriented (RM) or aligned (AM) silk scaffolds in the absence of ADSCs (Figure S2A,B, Supporting Information). Live‐dead staining was used to observe the cell viability of TSPCs. The cells in both RCM and ACM survived well (Figure 3C). No significant difference in cell viability was found between the ACM and RCM on day 1 and day 3 (Figure 3D). The proliferation of TSPCs was assessed using CCK‐8 assay at 1, 3, 5, and 7 days in both RCM and ACM. Results showed that proliferation was significantly increased in TSPCs treated with ACM at each time point as compared to RCM (Figure 3E). In addition, TSPCs in both ACM and RCM group proliferated faster than cells in RM and AM group (Figure S2C, Supporting Information). And no obvious differences were found between RM and AM group. ACM treatment also significantly up‐regulated the expression of the proliferation marker PCNA in mRNA level compared to the RCM treatment (Figure 3F). Moreover, the results of immunofluorescence showed that ACM induced significant increase of Ki67‐positive cells in TSPCs as compared to RCM group (57.71 ± 2.76 vs 18.74 ± 4.57, *p* < 0.001) (Figure 3G,H).

### 2.3 Effect of RCM and ACM on Tendinous Differentiation

**Figure 4:**
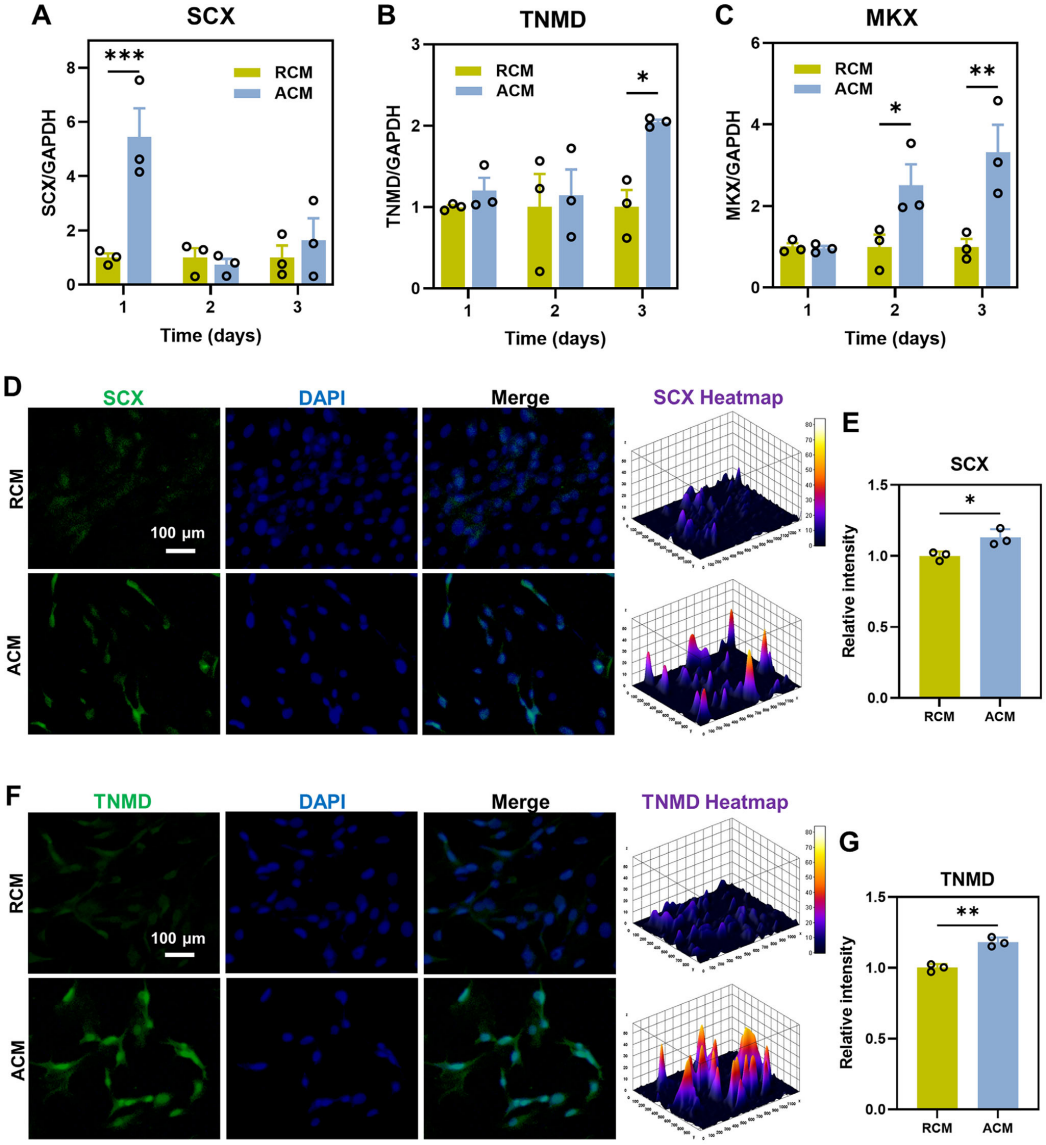
Effect of RCM and ACM on tendinous differentiation. A–C) TSPCs were cultured in RCM and ACM for 3 days. Gene expression of tendon markers was compared on days 1, 2, and 3. Levels of the RCM group were set as 1. Data are shown as Mean ± SEM, *n* = 3 technically independent samples for each group. D,F) The expression of SCX and TNMD were evaluated by immunofluorescence staining. Scale bar = 100 µm. E,G) Relative intensity of SCX and TNMD were quantified, *n* = 3 randomly‐selected microscopic images per group, *p < 0.05, **p < 0.01, ***p < 0.001.

The effect of RCM and ACM on tenogenic differentiation in TSPCs was assessed by measuring the expression levels of genes related to tendon differentiation after 1, 2, and 3 days of treatment. The expression of SCX, TNMD, and MKX genes was more significantly promoted in ACM group (**Figure** **4**A–C). More specifically, the expression of SCX was significantly higher in the ACM group at day 1 (5.44‐fold, *p* < 0.001) compared to the RCM group, but no significant difference was observed between the two groups at day 2 and 3. The gene expression levels of TNMD (2.04‐fold, *p* < 0.05) and MKX (3.32‐fold, *p* < 0.01) were significantly higher in the ACM group at day 3 as compared to RCM group. Similar to mRNA expression levels, the protein levels of SCX and TNMD were significantly up‐regulated in the ACM group compared with the RCM group (Figure 4D–G). Moreover, the expression of SCX and TNMD in both ACM and RCM group was significantly higher than that in RM and AM group (Figure S2D–G, Supporting Information). And no significant differences were found between RM and AM group. Therefore, RCM and ACM group were selected for the subsequent experiments.

### 2.4 Inflammatory Regulation Function of RCM and ACM

**Figure 5:**
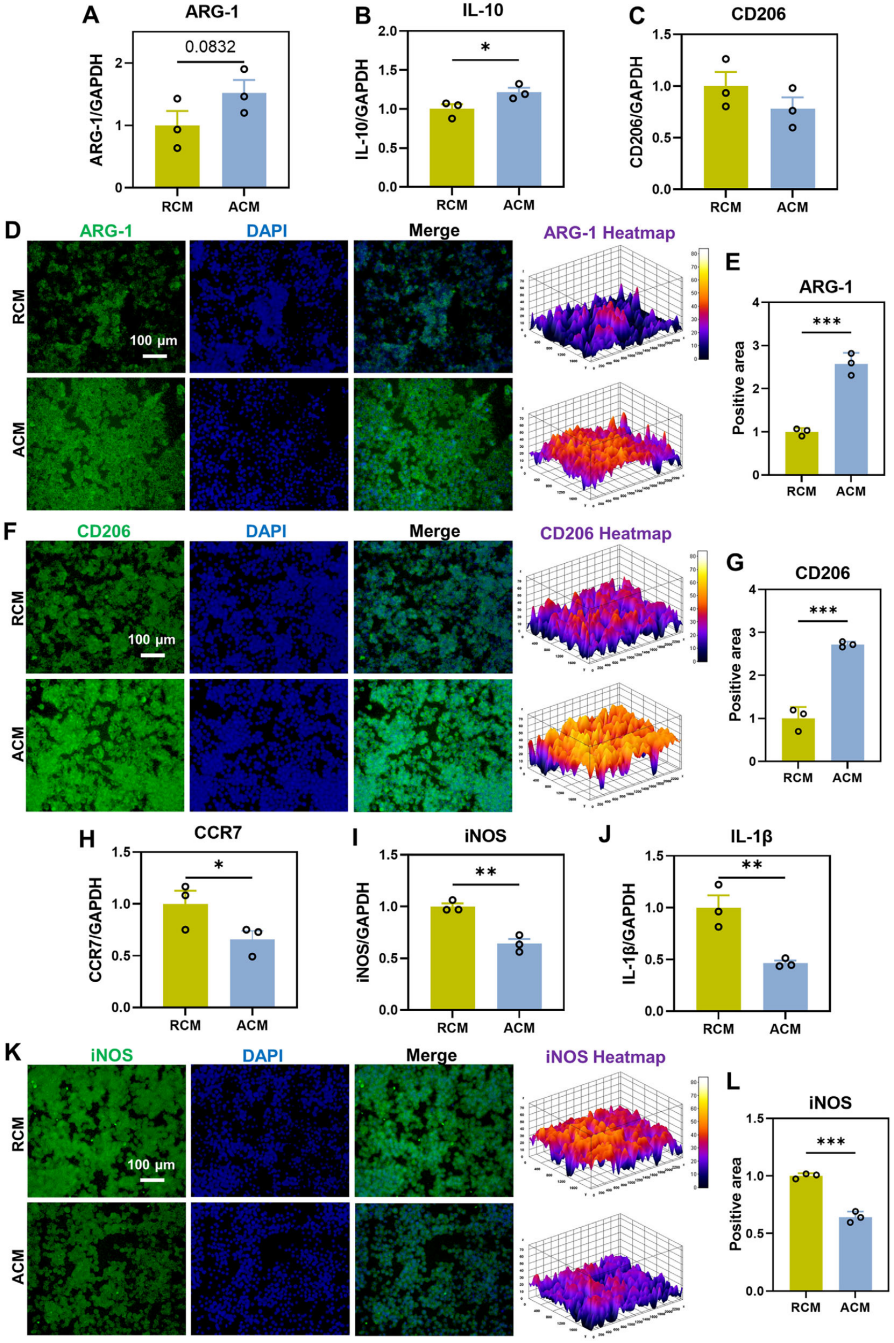
The effects of RCM and ACM on the polarization of macrophages. A–C) The expression of M2‐related anti‐inflammatory genes in RAW264.7 cultured in RCM and ACM for 2 days. Levels at the RCM group were set as 1, *n* = 3 technically independent samples for each group. D–G) The expression of ARG‐1 and CD206 were evaluated by immunofluorescence staining after 2 days, and the fluorescence positive area were quantified by ImageJ. Scale bar = 100 µm. *n* = 3 randomly‐selected microscopic images per group. H–J) The expression of M1‐related inflammatory genes. Levels at the RCM group were set as 1, *n* = 3 technically independent samples for each group. K,L) The expression of iNOS were evaluated by immunofluorescence staining after 2 days, and fluorescence positive area were quantified by ImageJ. Scale bar = 100 µm. *n* = 3 randomly‐selected microscopic images per group, *p < 0.05, **p < 0.01, ***p < 0.001.

To evaluate the regulatory effects of the ADSCs secretome on the polarization of macrophages, RCM and ACM were introduced to the cell culture of macrophages on TCP. Up‐regulation of M2‐related genes such as ARG‐1 (1.52 ± 0.36 vs 1.00 ± 0.40, *p* = 0.0832) and IL‐10 (1.22 ± 0.10 vs 1.00 ± 0.11, *p* < 0.05) was found in macrophages cultured in ACM as compared to in RCM (**Figure** **5**A,B). In contrast, the ACM group showed a down‐regulation of the M2‐related gene CD206 (0.78 ± 0.19 vs 1.00 ± 0.24) compared to RCM (Figure 5C), but the difference was not statistically significant. In the protein level, ARG‐1 (2.57 ± 0.26 vs 1.00 ± 0.09, *p* < 0.001) and CD206 (2.72 ± 0.06 vs 1.00 ± 0.26, *p* < 0.001) were significantly up‐regulated in the ACM group compared with the RCM group (Figure 5D–G; Figure S3A,B, Supporting Information). The expression of M1‐related genes was down‐regulated in the ACM group as compared to that in the RCM group, such as CCR7 (0.66 ± 0.14 vs 1.00 ± 0.22, *p* < 0.05), iNOS (0.64 ± 0.08 vs 1.00 ± 0.05, *p* < 0.01) and IL‐1β (0.46 ± 0.04 vs 1.00 ± 0.21, *p* < 0.01) (Figure 5H–J). In the protein level, iNOS (0.64 ± 0.05 vs 1.00 ± 0.02, *p* < 0.001) was significantly down‐regulated in the ACM group compared with the RCM group (Figure 5K,L; Figure S3C, Supporting Information).

### 2.5 ACM Effectively Promotes Tendon Regeneration In Vivo

#### 2.5.1 Histological Evaluation

**Figure 6:**
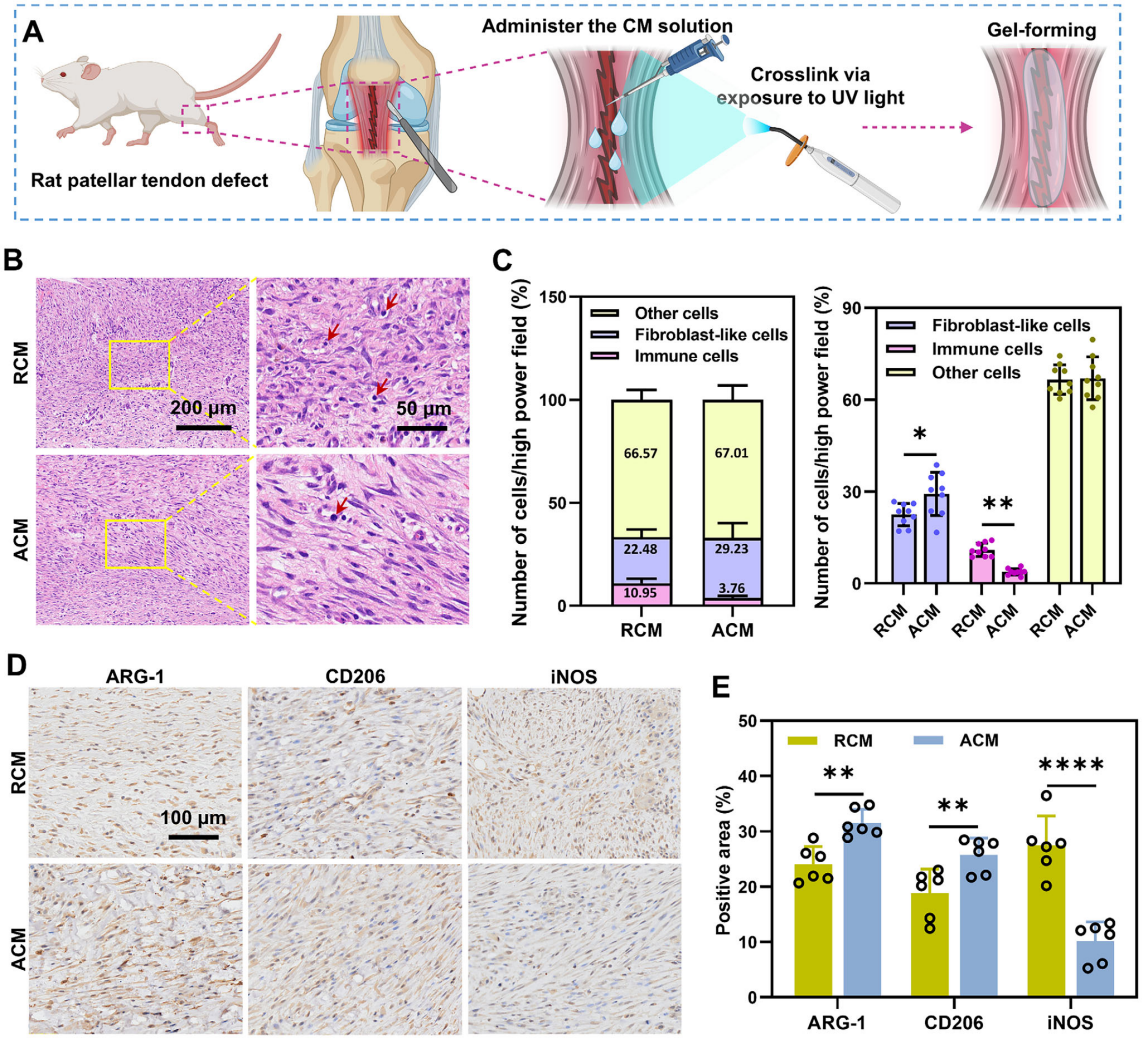
Histological examination and immunohistochemical staining of macrophage polarization markers in early stage of repaired tendons. A) Schematic diagram of in vivo experimental operation. The tendon defect is performed bilaterally. The figure was generated with BioRender (https://biorender.com/). B) H&E staining of patellar tendons from RCM and ACM groups at one week postoperatively. Red arrows indicate immune cells. Scale bar = 200 µm, 50 µm. C) The proportion rates of immune cells, fibroblast‐like cells, and other cells were quantified and compared. *n* = 9 randomly‐selected microscopic images per group, *p < 0.05, **p < 0.01. D) Immunohistochemical staining of macrophage polarization markers in repaired tendons. ARG‐1 and CD206 (M2, anti‐inflammatory), iNOS (M1, pro‐inflammatory). Scale bar = 100 µm. E) Quantifications of immunohistochemical stainings. *n* = 6 randomly‐selected microscopic images per group, **p < 0.01, ****p < 0.0001.

To test the effects of RCM and ACM on tendon regeneration in vivo, CMs were mixed with Silk Fibroin Methacryloyl (SilMA), added into the patellar tendon defect of rats and crosslinked with UV light to form hydrogel (**Figure** **6**A). H&E staining and immunohistochemistry staining was conducted at one week postoperatively. The ACM group had more fibroblast‐like cells and fewer immune cells compared to the RCM group, while the proportion of other cells tended to be similar between the two groups (Figure 6B,C). Immunohistochemical staining of the typical macrophage polarization markers ARG‐1, CD206 (M2, anti‐inflammatory), and iNOS (M1, inflammatory) were performed to evaluate whether the different CMs could influence the phenotype of macrophages during early stage of tendon repair. The ACM group showed a significantly higher expression of ARG‐1 (31.56 ± 2.46%), CD206 (25.76 ± 3.06%) and lower expression of iNOS (10.13 ± 3.52%) as compared to the RCM group (ARG‐1, 24.07 ± 3.19%; CD206, 18.84 ± 4.36%; iNOS, 27.47 ± 5.35%) (Figure 6D,E).

**Figure 7:**
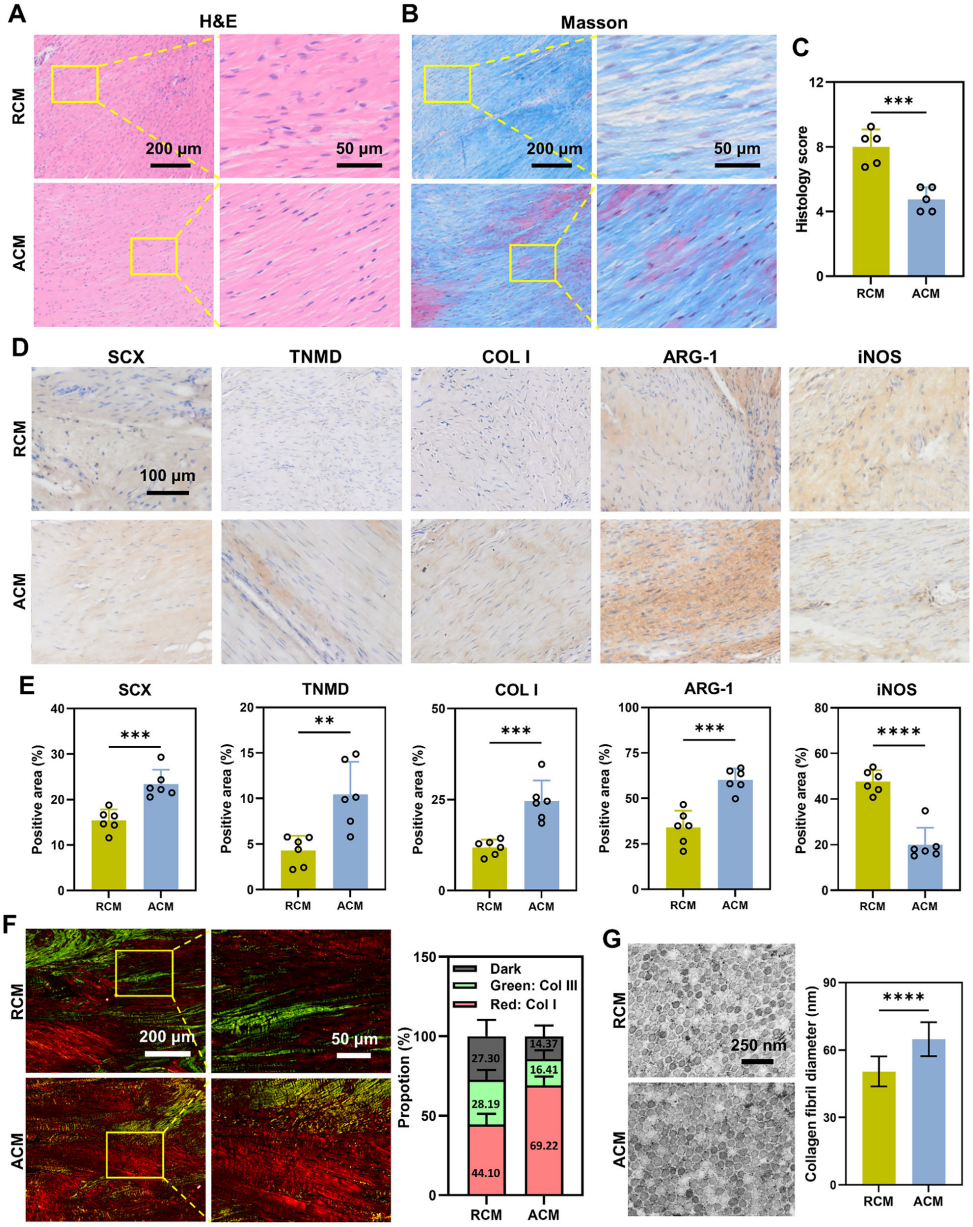
Histological examination and immunohistochemical staining of different markers in repaired tendons. H&E (A) and Masson's trichrome (B) staining of patellar tendons from RCM and ACM groups at 4 weeks postoperatively. Scale bar = 200 µm, 50 µm. C) Histological scoring of regenerated patellar tendon at 4 weeks postoperation. *n* = 5 randomly‐selected microscopic images per group, ***p < 0.001. D) Immunohistochemical staining of different markers in repaired tendons. Tendon‐related markers: SCX, TNMD, COL I; Macrophage polarization markers: ARG‐1 (M2, anti‐inflammatory), iNOS (M1, pro‐inflammatory). Scale bar = 100 µm. E) Quantifications of immunohistochemical stainings. *n* = 6 randomly‐selected microscopic images per group, **p < 0.01, ***p < 0.001, ****p < 0.0001. F) Picrosirius red‐staining of patellar tendons from RCM and ACM groups with polarized light. Scale bar = 200 µm, 50 µm. The proportion rates of collagen I (red or light orange area), collagen III fibers (green area), and dark area were quantified. *n* = 6 randomly‐selected microscopic images per group. G) TEM images of the collagen ultrastructure of RCM and ACM group. Scale bar = 250 nm. The collagen fibrils’ diameters were quantified. *n* = 100 randomly‐selected collagen fibrils per group, ****p < 0.0001.

After 4 weeks, histological examination using H&E staining (**Figure** **7**A) revealed that the histological morphology of ACM group was closer to that of normal tendon, with bundles of fibrous structures and spindle cellular nuclei. The Masson trichrome staining results revealed that more abundant and well‐organized denser collagen fibers were depositied in the ACM group compared to the RCM group (Figure 7B). Nevertheless, the collagen fibers observed in the ACM group were not fully packed, and there were still non‐spindle nuclei cells and inflammatory cells, suggesting that the regenerated tendons were not equivalent to normal tendons. The histological score of the ACM group was significantly better than the RCM group (4.25 ± 0.68 vs 8.00 ± 0.37, *p* < 0.001) based on factors including fiber structure, fiber arrangement, nuclear roundness, vascular numbers, inflammatory cell infiltration, and cell quantity (Figure 7C; Figure S4A, Supporting Information), indicating that ACM had a positive effect on tendon repair.

Immunohistochemistry staining was performed for tendon markers and macrophage polarization markers to evaluate the modulation of tendon repair and regeneration (Figure 7D,E; Figure S4B–E, Supporting Information). The results showed that the cells in the ACM group had significantly higher expression of SCX (23.41 ± 3.16%) and TNMD (10.42 ± 3.61%) than cells in the RCM group (SCX, 15.40 ± 2.46%; TNMD, 4.29 ± 1.61%), indicating the promotion effects of ACM on the expression of tendon markers. The main type of collagen found in tendons is COL I, and the ACM group showed significantly higher levels of COL I (24.59 ± 5.66%) compared to the RCM group (11.83 ± 2.14%). Increased type III collagen content occurs mostly in impaired healing of the extracellular matrix, collagen degeneration, and is a prominent feature of tendinopathy.^[^ ^39^ ^]^ From the immunohistochemistry staining, the ACM group showed a significantly lower expression of COL III (10.91 ± 1.52%) as compared to the RCM group (15.18 ± 2.48%) (Figure S4B,C, Supporting Information). Consistent with the changes from in vitro data, the ACM group showed a significantly higher ARG‐1 (60.13 ± 6.27%), CD206 (80.18 ± 8.26%) and lower iNOS (20.06 ± 7.39%) as compared to the RCM group (ARG‐1, 33.98 ± 9.26%; CD206, 67.17 ± 10.64%; iNOS, 47.67 ± 4.99%) (Figure 7D,E; Figure S4D,E, Supporting Information). The polarized light microscopy further showed that ACM group exhibited a higher ratio of collagen I (red or light orange area), and lower ratio of collagen III fibers (green area) than the RCM group (Figure 7F; Figure S4F, Supporting Information). TEM analysis (Figure 7G; Figure S4G, Supporting Information) revealed that the diameters of the collagen fibrils in ACM group (65.69 ± 7.05 nm) were significantly larger than those in RCM group (50.86 ± 7.36 nm).

#### 2.5.2 The Assessment of Tendon Function Recovery

**Figure 8:**
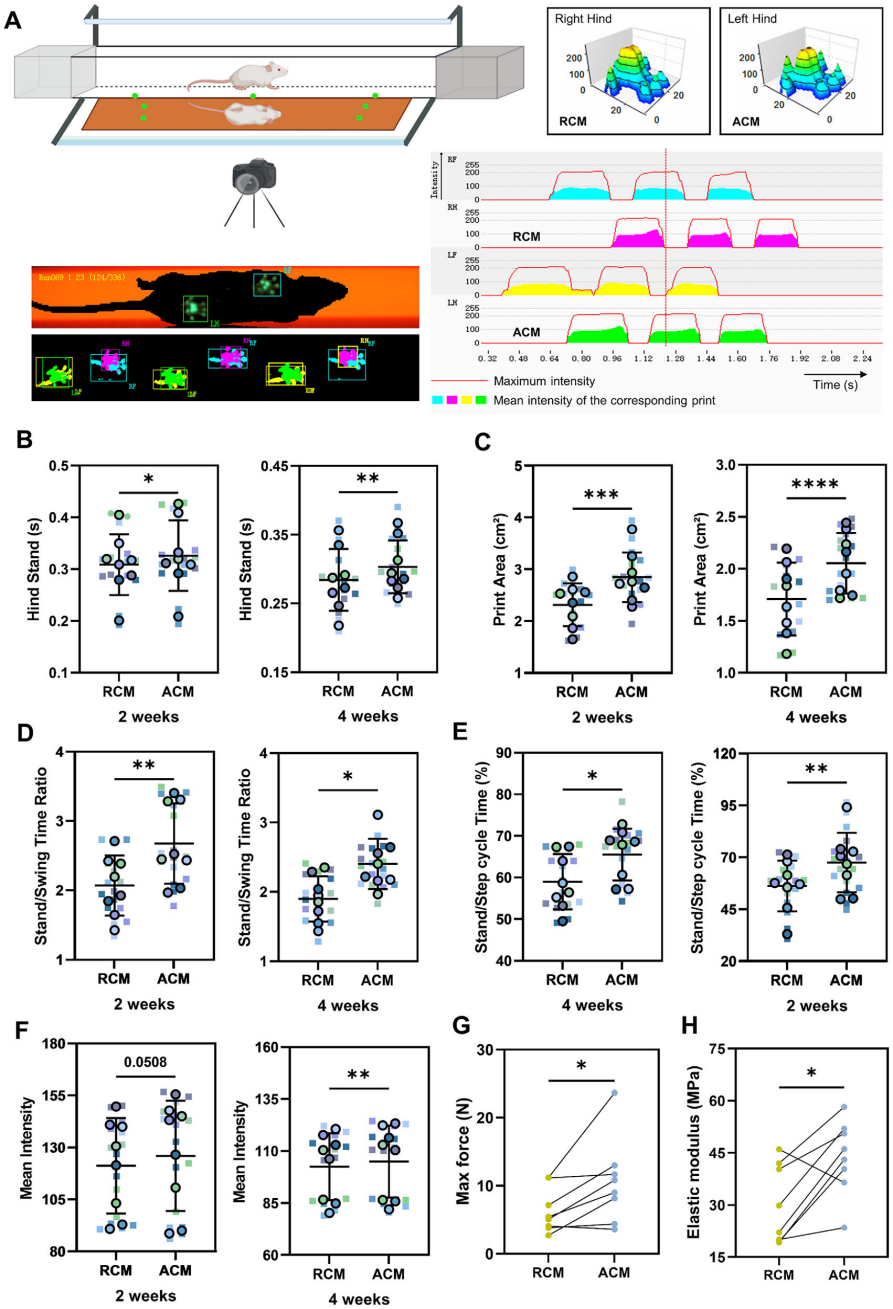
Gait analysis and biomechanical evaluation for the rats after being administered with RCM and ACM. A) Schematic diagram of gait analysis and representative gait screen shot, gait pattern, gait intensity, and 3D footprint intensity. The schematic diagram of gait analysis was generated with BioRender (https://biorender.com/). B) Quantification of hind stand in 2 weeks and 4 weeks. C) Quantification of hind print area in 2 weeks and 4 weeks. D) Quantification of hind stand/swing time ratio in 2 weeks and 4 weeks. E) Quantification of hind stand/step cycle time in 2 weeks and 4 weeks. F) Quantification of hind mean intensity in 2 weeks and 4 weeks. The data were analyzed by paired *t*‐test. RF right front, LF left front, RH right hind, LH left hind. *n* = 16 randomly‐selected results from 8 biological samples, *p < 0.05, **p < 0.01, ***p < 0.001, ****p < 0.0001 G) Comparison of max force between groups. *n* = 8 biologically independent samples, *p < 0.05. H) Comparison of mechanical tensile modulus between groups. *n* = 8 biologically independent samples, *p < 0.05.

To evaluate the tendon function recovery after patellar tendon defect, we performed gait analysis experiments at 2 and 4 weeks, and performed mechanical stretching experiments on the repaired tendons after 4 weeks. **Figures** **8**A S5A,B (Supporting Information) showed the representative gait screen shot, gait pattern, gait intensity, 3D footprint intensity, and football pattern. Rats were randomly administered with RCM in one of its hind legs and ACM in the other, and we compared the parameters indicating the left and right hind functions, including stand, print area, stand/swing time ratio, stand/step cycle time (%), and mean intensity. It was found that the values of standing time (Figure 8B), paw print area (Figure 8C), the stand/swing time ratio (Figure 8D), the stand/step cycle time (%) (Figure 8E), and the swing speed (Figure S5C, Supporting Information) of the hind legs were significantly higher in the ACM group at both the 2 weeks and 4 weeks points. The mean intensity at 2 weeks point did not show significant differences (*p* = 0.0508) between the two groups. While the mean intensity in ACM group was significantly improved as compared to RCM group at 4 weeks point (Figure 8F). The results showed that compared with the RCM group, the recovery of the tendon function in the ACM group was reflected in the extension of paw standing time and the increase of paw print area, stand/swing time ratio, stand/step cycle time (%), swing speed and the mean intensity. By the biomechanical evaluation experiment of the repaired tendons, we found that compared with RCM group, the max force, ultimate tensile strength and mechanical tensile modulus of tendons in ACM group were all significantly improved (Figure 8G,H; Figure S5D,E, Supporting Information).

### 2.6 Proteomic Analysis Shows the Inherent Difference Between RCM and ACM

**Figure 9:**
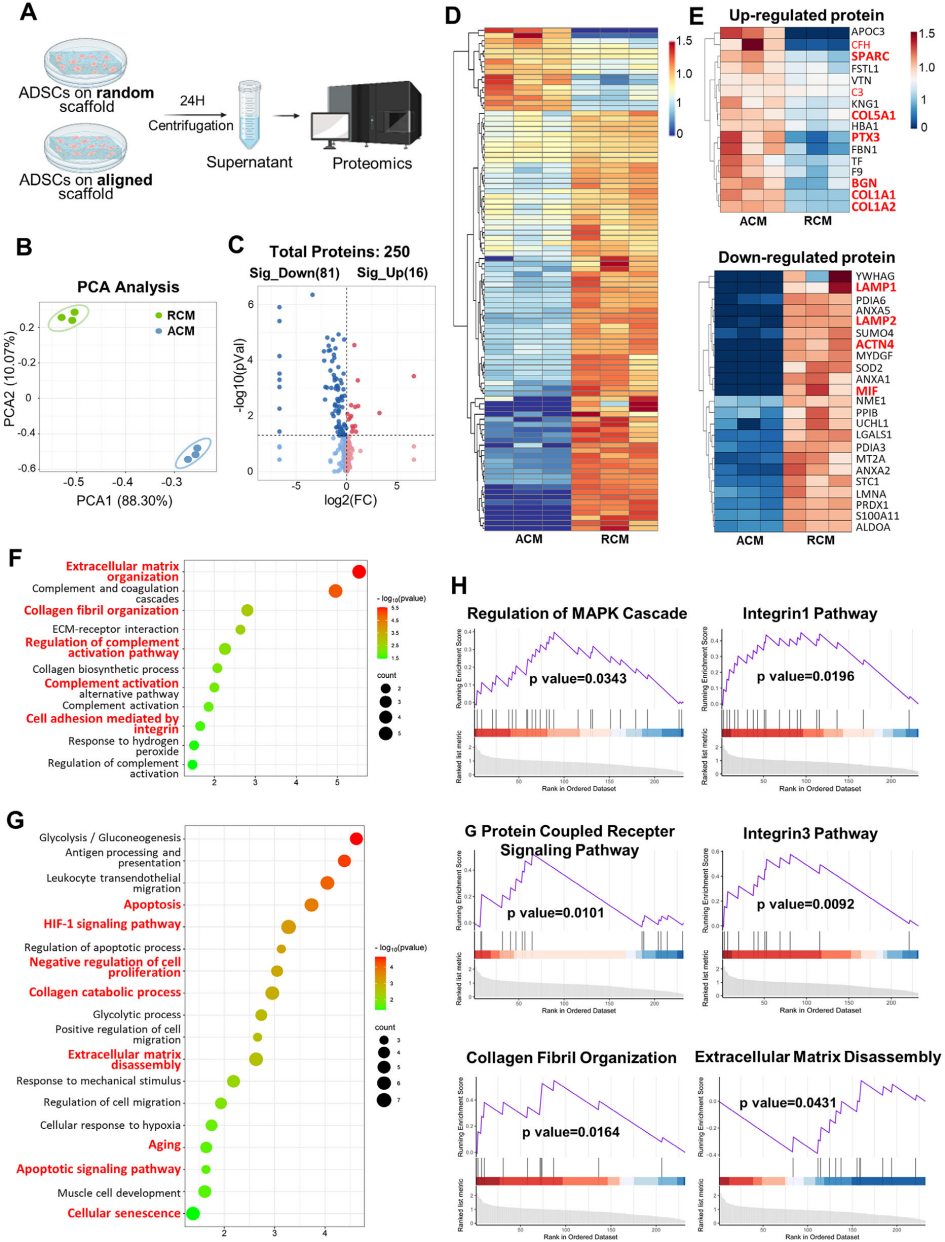
Proteomic analysis of ACM and RCM. A) Schematic diagram of the proteomic profiling of ACM and RCM (*n* = 3 independent experimental units (EUs)). The figure was generated with BioRender (https://biorender.com/). B) PCA plot of proteomic data in ACM and RCM groups. C) Differentially‐expression analysis of secretome between ACM and RCM. Significantly up‐ or down‐regulated proteins are in red or blue. D) Heatmap profiling the hierarchical cluster analysis of secretome of ACM and RCM. E) Heatmaps of the all up‐regulated and the top 23 down‐regulated proteins in ACM. F,G) Gene ontology (GO) analysis of the differentially‐expressed proteins. H) Gene set enrichment analysis (GSEA) of differentially‐expressed proteins.

To elucidate the mechanism of ACM in promoting tendon repair, we analyzed the components of ADSCs secretome on the different topographic structure by proteomic analysis (**Figure** **9**A). The samples were examined and 250 proteins were identified. The PCA plot showed that the samples of each group were assembled into a cluster (Figure 9B). Differentially‐expression protein analysis indicated that 16 proteins were significantly increased in the ACM group, while 81 proteins were reduced, compared to the RCM group (Figure 9C). The heatmap of all identified proteins showed a difference in the protein expression after cluster analysis (Figure 9D). Specially, the ACM group had significantly higher expression of ECM‐related proteins, including SPARC, COL5A1, BGN, COL1A1, and COL1A2, and anti‐inflammatory‐related protein, including PTX3. While the RCM group showed a notably increased expression of proinflammatory‐related protein, including MIF, and apoptosis‐related protein, including LAMP1, LAMP2, and ACTN4. The protein interaction network for the up‐regulated and down‐regulated proteins, respectively from DEPs were screened using CytoHubba in Cytoscape (Figure S6A,B, Supporting Information). Gene ontology (GO) analysis revealed the up‐regulated members of secretome in ACM group related to extracellular matrix organization, collagen fibril organization, cell adhesion, and complement activation process (Figure 9F). The down‐regulated proteins in ACM group were related to several crucial biological processes in apotosis, negative regulation of cell proliferation, extracellular matrix disassembly, aging, cellular senescence, and pathways of HIF signaling and apoptotic signaling, which can explain why enhanced tendon regeneration was observed in the ACM group as compared to the RCM group (Figure 9G). Meanwhile, Gene set enrichment analysis (GSEA) also showed that more functional/molecular pathways were activated in ACM group, including MAPK cascade, Integrin1/3 pathway, and G protein coupled recepter (GPCR) signaling pathway (Figure 9H).

**Figure 10:**
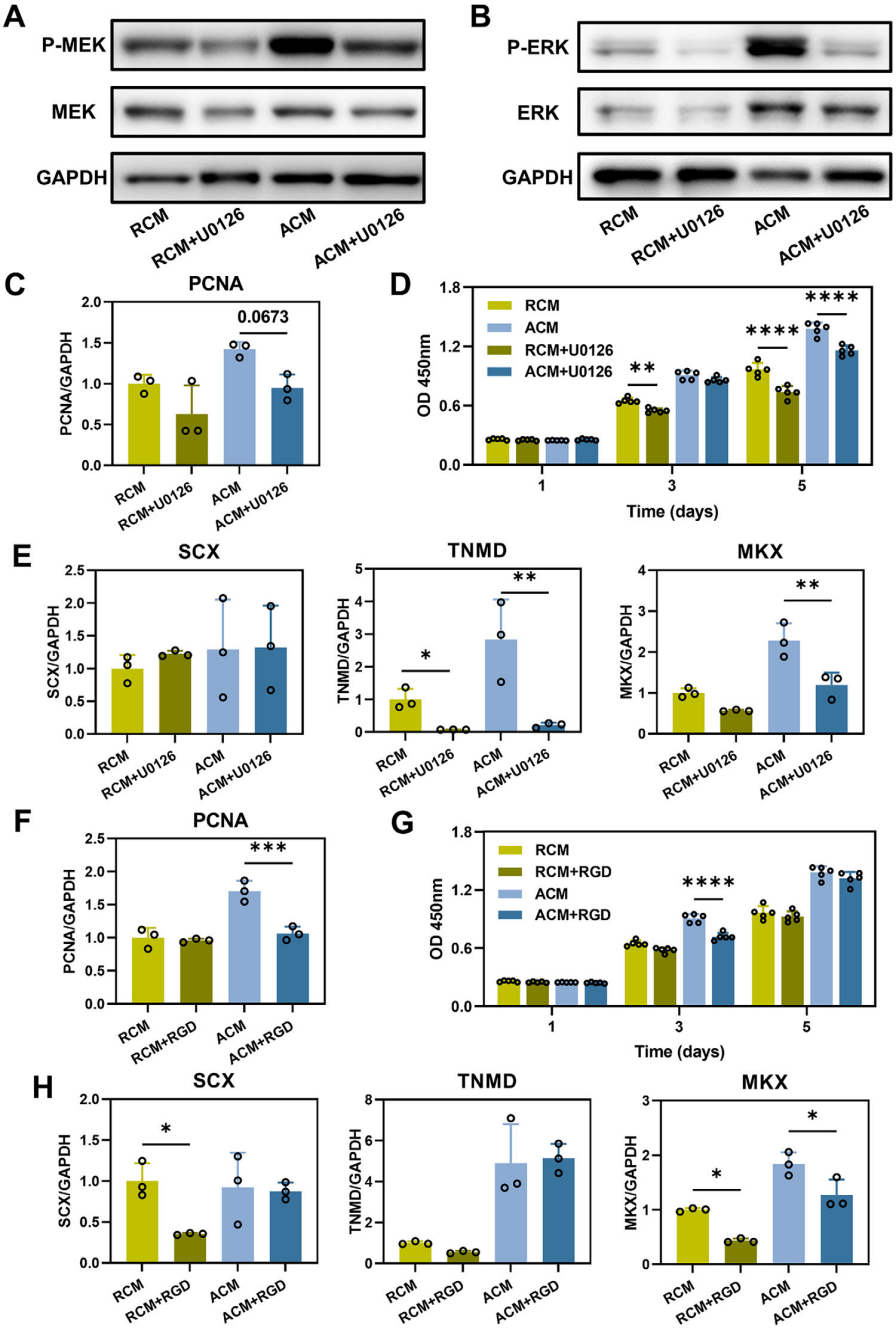
ACM improves cell proliferation and tendon differentiation of TSPCs by activating MAPK and Integrin pathways. A) Western blot of p‐MEK and MEK in TSPCs after treatment of RCM, RCM+U0126, ACM, or ACM+U0126 for 18 h. B) Western blot of p‐ERK, ERK in TSPCs after treatment of RCM, RCM+U0126, ACM, or ACM+U0126 treatment for 18 h. C) Gene expression of PCNA in different groups at day 3. D) Proliferation of TSPCs measured by CCK‐8 at 1, 3, and 5 days, *n* = 5 technically independent samples for each group, **p < 0.01 ****p < 0.0001. E) Gene expression of SCX, TNMD, and MKX in different groups at day 3. Levels at the RCM group were set as 1. Data were shown as Mean ± SD, *n* = 3 technically independent samples for each group, *p < 0.05, **p < 0.01. F) Gene expression of PCNA in different groups at day 3, ***p < 0.001. G) Proliferation of TSPCs measured by CCK‐8 at 1, 3, and 5 days, *n* = 5 technically independent samples for each group, ****p < 0.0001. H) Gene expression of SCX, TNMD, and MKX in different groups at day 3. Levels at the RCM group were set as 1. Data were shown as Mean ± SD, *n* = 3 technically independent samples for each group, *p < 0.05.

To verify and evaluate the effects of differential activation of MAPK pathway between ADSCs secretome from different topology structures, we first detected the expression levels of representative p‐MEK, MEK, p‐ERK, and ERK^[^ ^40^ ^]^ in TSPCs treated with RCM and ACM. Our results revealed that the levels of p‐MEK/MEK and p‐ERK/ERK in the ACM group were significantly higher than those in the RCM group (Figure S6C, Supporting Information), indicating the activation of MAPK signaling pathway in TSPCs after ACM treatment. To evaluate the role of MAPK signaling pathway, TSPCs were treated with CMs and U0126 (a selective MEK/ERK pathway inhibitor). Both levels of p‐MEK and p‐ERK were decreased after the treatment of U0126 (**Figure** **10**A,B). Meanwhile, the expression of proliferation marker PCNA was downregulated (Figure 10C), as well as the cell proliferation capacity shown by CCK‐8 assay (Figure 10D). Likewise, the expression levels of genes related to tendon differentiation (TNMD and MKX) were significantly lower in the ACM+U0126 and RCM+U0126 groups compared to the ACM and RCM groups respectively (Figure 10E). These data indicates that the ADSCs secretome in aligned scaffold could activate MAPK signaling pathway which contributes to the promotion effects of ACM on tendon regeneration.

To evaluate Integrin 1/3 pathway, we detected the gene expression of ITGA1, ITGA11, and ITGB1^[^ ^41^ ^]^ by qPCR. Our results revealed that the expression levels of ITGA1 (1.34‐fold), ITGA11 (1.84‐fold), and ITGB1 (1.84‐fold) in the ACM group were higher as compared to those in the RCM group (Figure S6D, Supporting Information). TSPCs were further treated with CMs and RGD peptides (an inhibitor of integrin signaling). As a result, the expression of PCNA was inhibited in the ACM+RGD group as compared to the ACM group (Figure 10F). And the cell proliferation capacity was downregulated too (Figure 10G). Similar as the treatment of U0126, the expression levels of tendon markers were decreased after the treatment of RGD (Figure 10H). These data indicate that Integrin 1/3 pathway was involved in the differential effects of ACM and RCM on tendon regeneration.

GPCRs can modulate receptor internalization and desensitization through β‐arrestin upon activation, activate various downstream signaling pathways, thereby mediating and regulating multiply vital biological activities.^[^ ^42^ ^]^ To verify the differential activation of GPCR signaling pathway in TSPCs due to ADSCs secretome from different topology structures, western blot was conducted to detect the protein expression of β‐arrestin. Our results revealed that the level of β‐arrestin/GAPDH in the ACM group was significantly higher than that in the RCM group (Figure S6E, Supporting Information).

## 3 Discussion

This study aimed to evaluate the influence of topology‐based secretomic differentiation on tendon repair. The results indicated that the secretome obtained from ADSCs cultured on the aligned silk scaffold (ACM) had a superior impact on cell migration, proliferation, and tendinous differentiation of TSPCs compared to the secretome derived from ADSCs cultured on a randomly oriented scaffold (RCM). Additionally, the use of ACM was found to promote M2‐type macrophages instead of M1‐type, suggesting anti‐inflammatory effect on tendon repair. Compared with the RCM group, the ACM exhibited the capacity to accelerate tendon healing by improved reconstruction of tendon histomorphology and restoration of mechanical strength. Moreover, proteomic analysis indicated that the secretome from ACM could enhance extracellular matrix organization, and limit apoptotic pathways. These findings underscore the influence of scaffold topology on the secretome and paracrine effects of ADSCs in tendon repair, with potential implications for cell‐free therapies not only in tendon regeneration but also in other types of tissue injuries.

Currently, cell‐free therapy is increasingly recognized as an important approach in regenerative medicine for treating tissue injuries including tendons. Studies have shown that MSCs‐CM is a competitive alternative to the parent cells due to their similar beneficial effects^[^ ^43^ ^]^ and can mediate the paracrine effects of the MSCs.^[^ ^44^ ^]^ Compared to traditional cell injection or implantation, cell‐free therapy using CM without cell components but containing secreted products has several therapeutic advantages in tissue regeneration: 1) MSCs‐CM can be isolated from immortalized MSCs, enabling their application in cell therapy, which is not applicable to the immortalized cells themselves.^[^ ^45^ ^]^ 2) MSCs‐CM is less immunogenic compared to whole cell therapy, making it more applicable in different individuals,^[^ ^46^ ^]^ considering immune rejection of allogeneic mesenchymal stem cell transplantation.^[^ ^47^ ^]^ Graft rejection was mainly caused by the mismatch of major histocompatibility complex (MHC) molecules (classes I and II) on the cell membrane.^[^ ^48^ ^]^ The lack of cell membrane epitopes in MSCs‐CM makes it a promising candidate for allogeneic transplantation, with potential for wide applicability in various clinical scenarios. 3) The quantification and storage of the optimal concentration of CM for future applications can be easily achieved,^[^ ^49^ ^]^ but additional experimental studies are required to determine the ideal concentration suitable for various specific applications.

Numerous studies have shown that the differentiation and tissue regeneration ability of MSCs are significantly affected by the cell microenvironment, and the physical regulation of topological structure can change the paracrine signaling of cells to influence tissue repair.^[^ ^32^, ^37^, ^50^ ^]^ Native ECM of tendon is composed of arranged collagen fibers. Previous study have shown that ordered orientation of scaffold structure can promote tendon differentiation and regeneration.^[^ ^51^ ^]^ However, the effect of the ordered structure on the cell secretome deserves to be investigated. Here, directional freeze‐drying was applied to obtain aligned topographical structure. Under the electron microscope, we observed that the pseudopods of ADSCs were expanded and bound to the surface of random silk scaffolds, while spindle‐like cells were resting in the gaps of aligned silk scaffold loosely (Figure 2G,H,K,L). Therefore, topography may regulate the secretome by affecting cell morphology. Further evaluations found that ACM promoted cell migration and proliferation, tenogenic differentiation, and regeneration (Figures 3, 4, 7, and 8). Kadir et al.^[^ ^37^ ^]^ reported that MSCs paracrine effects in cartilage repair differed when cultured on either aligned or random fiber orientation, suggesting the existence of a topographically dependent secretome of MSCs. Our study shows that the topology structure affected the ADSCs’ secretome and its potential for tendon remodeling and regeneration. To explore the regulation of scaffold structure on cell secretome, we performed proteomic analysis and found differentially‐secreted proteins with different scaffold. GO and GSEA analysis indicated that increased proteins in ACM were related to cell adhesion, and that reduced proteins were related to negative regulation of cell proliferation, apoptosis, aging, and cellular senescence (Figure 9F,G). Besides, the increased proteins in ACM were related to biological processes in extracellular matrix organization, collagen fibril organization, and the reduced proteins were related to biological processes in extracellular matrix disassembly (Figure 9F,G). These effects account for the enhanced tendon regeneration in the ACM group as compared to the RCM group.

Collagen deposition in extracellular matrix was considered a vital step in tendon regeneration.^[^ ^52^ ^]^ Previous reports showed that SPARC was a key extracellular matrix protein essential for tendon tissue maturation and homeostasis.^[^ ^53^ ^]^ In this study, SPARC protein was highly up‐regulated in ACM group consistent with the high levels of COL5A1, BGN, COL1A1, and COL1A2 (Figure 9E). Therefore, we speculated that topological structure can build a benign microenvironment to up‐regulate the secretion of tendon‐related cell matrix protein, which is conducive to tendon repair and regeneration. Previous study has shown that the MAPK/p38/Cyclin D1 pathway plays an important role in tendon healing, which is involved in the enhanced effect of PDGF on cell proliferation, stem cell tenogenesis, and ECM deposition.^[^ ^54^ ^]^ In addition, integrin has been shown to play a role in the upregulated tenogenic differentiation of TSPCs, as the expression of integrins, cellular matrix proteins, tendon‐specific genes, and matrix metalloproteinases (MMPs) were all increased after mechanical activation.^[^ ^55^ ^]^ In the GSEA, western blot, and qPCR results of our study, ACM upregulated the MAPK and Integrin 1/3 pathways (Figure 9H; Figure S6C,D, Supporting Information), which aligns well with the improved histological and functional repair of tendons in the ACM group. GPCRs transmit extracellular stimuli to achieve specific cellular functions. Although cells express numerous types of GPCRs, they all signal through a limited number of second messengers, such as cAMP.^[^ ^56^ ^]^ Arrestins comprise a small protein family important for GPCR signaling. GPCR phosphorylation is required for the recruitment and activation of arrestin, which decouples the G protein from the receptor and triggers receptor internalization and initiation of the arrestin‐mediated signaling cascade.^[^ ^57^ ^]^ Our western blot results revealed that the relative β‐arrestin/GAPDH in the ACM group was significantly higher than that in the RCM group (Figure S6E, Supporting Information), indicating the differential activation of GPCR pathway in TSPCs from RCM or ACM treatment. Previous study has confirmed that the activation of cAMP/PKA pathway can inhibit fat infiltration, promote tendon‐to‐bone healing, improve biomechanical properties, and reduce the risk of injury rupture.^[^ ^58^ ^]^ GATA6 expression activates the cAMP/PKA pathway, leading to the formation of the oriented tendon‐to‐bone junction structure, increased matrix deposition, enhanced continuity of collagen fibers, and elevated expression of collagen I. The G protein coupled receptor pathway was enriched in ACM group (Figure 9H), and greater mechanical strength of repaired tendons was found in the ACM group (Figure 8G,H). These results indicate that parallel structure can up‐regulate the secretion of tendon relevant ECM proteins and activate stroma protein‐related pathways to promote tendon repair.

The process of tissue regeneration was influenced by the immune system. We found that PTX3 was significantly up‐regulated, whereas SOD2 down‐regulated in ACM group (Figure 9E). Similarly, previous study has shown that the high expression of PTX3 promoted the activation of M2 macrophages.^[^ ^59^ ^]^ PTX3 modulated the expression of polarization markers in macrophage and monocyte in a similar way as IL‐10, suggesting that it may enhance the acquisition of an M2 macrophage phenotype. PTX3 also promoted cell migration and induced upregulation of extracellular matrix genes such as Col 1A1. In contrast, the increased SOD2 were associated with macrophages pro‐inflammatory (M1) polarization.^[^ ^60^ ^]^ Consistently, we observed that ACM in our study are more likely to promote polarization of macrophages to M2 type (Figure 5A–G in vitro, Figure 6D,E, and 7D–E in vivo), while macrophages tended to be more M1 polarized in RCM group (Figure 5H–L in vitro, Figure 6D,E, and 7D,E in vivo). Therefore, it can be interpreted that topographical structure plays a role in the secretome of ADSCs for macrophage polarization and tendon repair. The activation of complement was highly correlated with the secreted proteins of ACM, the most obvious of which is the significant elevation of the complement H factor (CFH) protein (Figure 9E). Previous study showed that CFH controls intracellular C3 levels of macrophages in a cell‐autonomous manner and negatively regulates consumption of complement component 3 (C3), thereby restricting the complement activation,^[^ ^61^ ^]^ improve TNF‐α‐induced inflammation in rheumatoid joint inflammation,^[^ ^62^ ^]^ and inhibit vascular endothelial cell migration and anti‐angiogenesis in tumor cells.^[^ ^63^ ^]^ This is consistent with our observation of lower vascularity score in repaired tendons in the ACM group (Figure S4A, Supporting Information). Study has demonstrated that PTX3 can interact with CFH to downregulate the alternative complement pathway.^[^ ^64^ ^]^ This interaction promotes CFH recruitment to the PTX3 surface, thereby preventing excessive complement activation. These properties may cause CFH to play an anti‐inflammatory role in tendon repair, inhibit tendon vascularization, and achieve a better tendon repair effect. Nevertheless, few studies directly investigated the effect and function of complement activation in tendon tissue repair, which is worth to be further investigated in the future.

**Figure 11:**
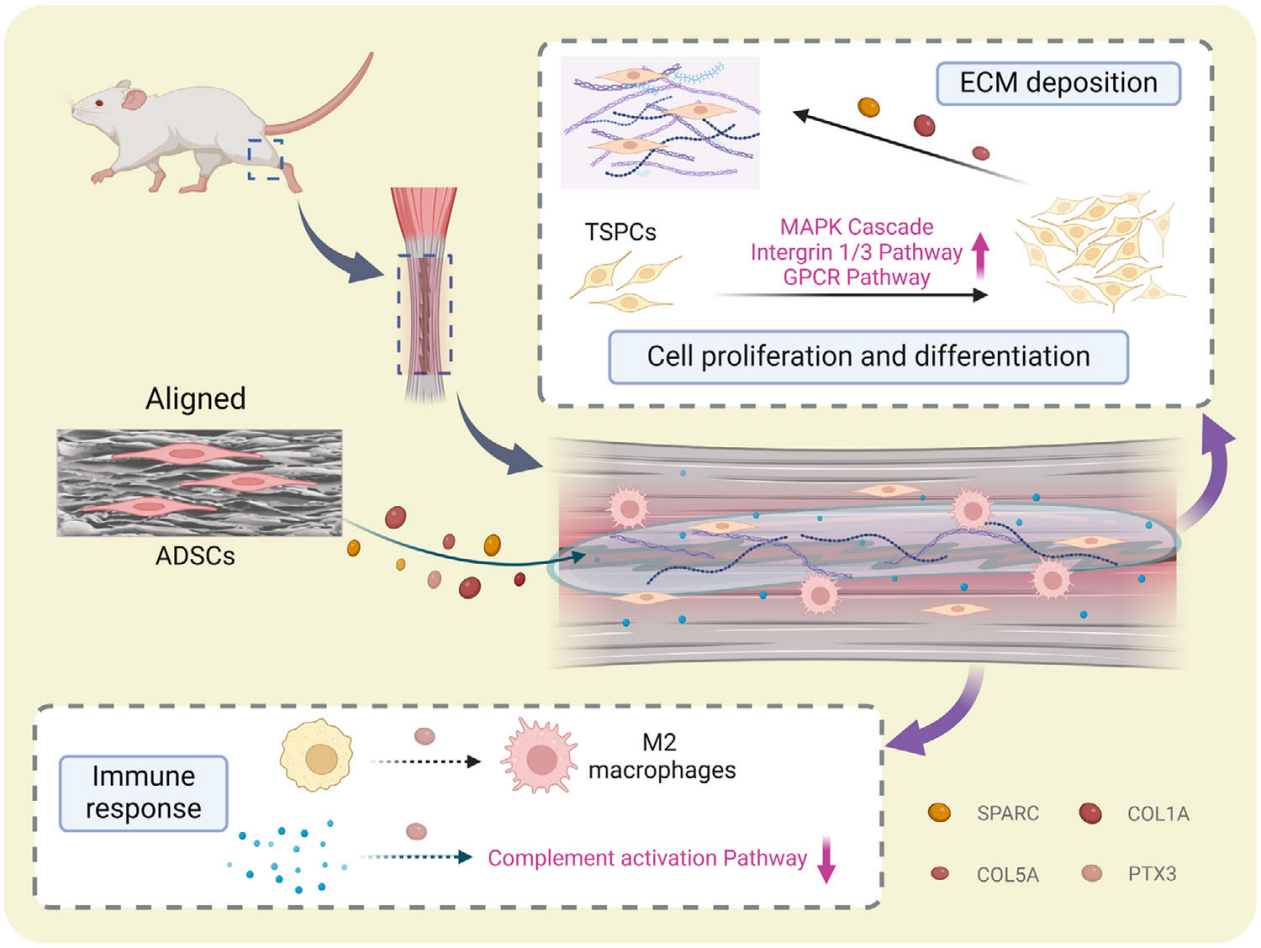
Schematic illustration of topology structure in promoting tendon regeneration by potentiating the paracrine effects of ADSC secretome. The figure was generated with BioRender (https://biorender.com/).

Our in vitro and in vivo results together suggest that topographic structure can influence the secreted proteins of ADSCs to regulate tendon regeneration. The ADSCs secretome on ordered structure scaffold can via paracrine signaling promote the cell proliferation, tenogenic differentiation, ECM deposition, anti‐inflammatory phenotype polarization of macrophages, and restrict complement activation. On the random oriented scaffolds, ADSCs generated apoptosis‐related proteins and macrophage pro‐inflammatory phenotypic polarization proteins leading to inferior effect on tendon repair (**Figure** **11**). Physical structure can affect the secretion profile of ADSCs and regulate tissue regeneration through corresponding pathways. The aligned structure mainly promotes the secretion of CFH, SPARC, C3, PTX3, collagen, and other proteins by ADSCs through activation of Integrin1, Integrin3, MAPK cascade reaction, and G protein‐coupled receptor signaling pathway. The random structure promotes LAMP1, LAMP2, ACTN4 protein secretion of ADSCs and HIF‐1 signaling pathway, which promotes collagen catabolic process, ECM disassembly, and negatively regulates the cell proliferation. Therefore, the ordered structure can enhance the secretion of more positive regulatory proteins by ADSCs, which has great potential effect on tendon regeneration.

However, there are several limitations in our study. First, MSCs derived from different tissues have different secretome. Although ADSCs and ADSCs‐CM have advantages, direct comparisons with other sources of MSCs (such as BMSCs, and TSPCs) in tendon repair are needed. Second, how the topographic properties regulate the ADSCs secretion remains to be explored in detail. Due to the technical limitation, the aligned scaffolds prepared in this study are not ideal. Completely parallel scaffolds, prepared by 3D printing or other advanced technologies would be valuable for further exploring the impact of topography on cellular secretion. Lastly, we used macrophages to reflect the tendon immune‐related activity. The immunomodulatory role of humoral immunity, cellular immunity, and complement activation regulation by ADSC‐CM for tendon regeneration remains to be further elucidated.

## 4 Conclusion 

In this study, we investigated the effect and mechanism of topography on the secretome of ADSCs for tendon regeneration. ADSCs cultured on aligned scaffold resulted in a secretome (ACM) that promoted TSPCs migration, cell proliferation, and tendon differentiation, as well as enhanced tendon repair with recovery of tissue structure and motor ability in vivo. Furthermore, the use of ACM resulted in the alteration of macrophage polarization toward the anti‐inflammatory M2 phenotype, and reduced the local inflammatory response in vivo. The proteomic analysis found that the aligned structure can enhance the secretion of more positive regulatory proteins (SPARC, COL1, COL5, PTX3) by ADSCs, which may have a significant impact on tendon regeneration. Further evaluation indicated that the secretome of ADSCs regulates extracellular matrix organization, cell proliferation and differentiation through differential activation of the MAPK, GPCR, and Integrin1/3 pathways. These findings shed light on a novel aspect of topographic structure and cellular interaction, with potential implications for cell‐free regenerative medicine in tendon.

## 5 Experimental Section

### Cell Culture

ADSCs were supplied from Genesis Stem‐cell Co.LTD (SWZ20110523). TSPCs were obtained from Achilles tendon tissues of adult Sprague Dawley (SD) rats as previously described.^[^ ^65^ ^]^ A low glucose DMEM (Gibco, C11885500) containing 10% fetal bovine serum (FBS, Wisent, 86550011) and 1% streptomycin‐amphotericin B‐penicillin (Gibco, 15240062) was used for cell culture. Cells were incubated at 37 °C, 5% CO_2_ with medium changed every 2–3 days. At 80–90% confluency, cells were passaged by trypsinization. ADSCs were expanded to passages 3–5 for further assays. TSPCs were used between passages 4–6. The macrophages (RAW 264.7 cells) were kindly provided by Prof. Wang from Southeast University, China, and were cultured in high glucose DMEM (Wisent, 319‐005‐CL) containing 10% FBS and 1% streptomycin‐amphotericin B‐penicillin.

### Identification and Multi‐Lineage Differentiation Potency of ADSCs

ADSCs were subjected to flow cytometry to assess their phenotypic characteristics. The levels of CD73 (Proteintech, FITC‐65162), CD90 (BioLegend, 328107), CD105 (BioLegend, 323203), CD34 (BioLegend, 343603), CD11b (Proteintech, FITC‐65116), CD19 (Proteintech, APC‐65110) and CD45 (Proteintech, APC‐65064) were evaluated. ADSCs were seeded in a 24‐well plate (NEST, 702001, 5 × 10^3^ cells per well for osteo‐, chondro‐lineage, and 2 × 10^4^ cells per well for adipose‐lineage) for multi‐lineage differentiation test as previously described.^[^ ^65^ ^]^ To induce osteogenic differentiation, cells were cultured in specific induction medium for one week and stained with alkaline phosphatase (ALP) (Beyotime, C3206). The osteoinductive medium consists of high‐glucose DMEM, 10% FBS, 1% streptomycin‐amphotericin B‐penicillin, 10 mm β‐Glycerophosphate disodium salt hydrate (Sigma, G5422), 10 nm dexamethasone (Sigma, D4902), and 50 µg mL^−1^ ascorbic acid (Sigma, A1968). To induce chondrogenic differentiation, cells were cultured in specific induction medium for 2 weeks and stained with Alcian Blue (Macklin, A801642). The chondrogenic medium consists of high‐glucose DMEM, 1% Insulin‐Transferrin Selenium (ITS, Gibco, 41400045), 1 mm sodium pyruvate (Gibco, 11360070), 50 µg mL^−1^ ascorbic acid, and 10 ng mL^−1^ TGF‐β1 (PeproTech, 100‐21). To induce adipogenic differentiation, cells were cultured in specific induction medium for two weeks and stained with Oil Red O (Solarbio, G1260). The adipoinductive medium consists of high glucose DMEM with 10% FBS, 1% streptomycin‐amphotericin B‐penicillin, 1 mm dexamethasone, 10 µg mL^−1^ insulin (Sigma, I9278), and 0.5 mm isobutylxanthine (Sigma, I5879). The induction solutions were changed every 2 days.

### Fabrication and Characterization of Aligned and Random Silk Scaffold

The preparation of aligned and random silk scaffold followed the same protocol as in the previous studies.^[^ ^66^, ^67^ ^]^ Silk fibroin was extracted from silk by elution, dissolution, and dialysis. The 6% silk solution was placed in a polyethylene tube (diameter 12 mm × height 52 mm). To prepare the aligned scaffold, the bottom of the tube was placed on a pre‐cooled −80 °C aluminum plate and frozen in a fixed direction. To prepare the random scaffold, the tube with silk solution was placed in a foam plate and frozen from all directions to the center of the solution. After fully freezing, the scaffolds were lyophilized (Boyikang, FD‐1A‐50), treated with 90% methanol (v/v, Sinopharm), and lyophilized again. Finally, scaffolds were cut to the appropriate size for experiments. To observe the surface structure of the scaffolds, they were mounted on stubs, coated with gold, and observed under a Zeiss EVO 18 scanning electron microscopy (SEM, Carl‐Zeiss). SEM images of the freeze‐dried scaffolds were used to quantify the pore alignment. “OrientationJ” and “Directionality” plugins, from Fiji were used to create the color maps of the pore orientation and to generate data to plot the directionality curves, respectively.^[^ ^68^ ^]^

### Preparation of CMs

To obtain RCM and ACM, ADSCs (6 × 10^5^ cells) were seeded on randomly oriented or aligned silk scaffolds. Similar as previous studies,^[^ ^69^, ^70^, ^71^ ^]^ after 24 h, a serum‐free high‐glucose DMEM was added. CMs were collected after 24 h, centrifuged at 1500 × rpm for 5 min, followed by 1800 × rpm for 10 min to remove dead cells and cellular debris, and sterilized with a 0.45 µm syringe filter (Millex‐HV Syringe Filter, SLHV033NK). To obtain RM and AM, the same volume of serum‐free high‐glucose DMEM was collected from randomly oriented or aligned silk scaffolds without ADSCs cultured after 24h. All the CMs were stored at ‐20 °C and used within one week to maintain their biological activity.^[^ ^72^, ^73^ ^]^ For each single experiment, the RCM and ACM used were derived from the same donor and same batch of ADSC cultures, and the experiment was successfully repeated using other donor‐derived ADSCs. To evaluate the effect of CMs on the tenogenic differentiation of TSPCs, cells were cultured on tissue culture plastic (TCP) and treated with CMs supplemented with 10% FBS, 1% streptomycin‐amphotericin B‐penicillin, and 0.25% ascorbic acid. The medium was changed every second day. To evaluate the effect of CMs on the cell polarization of the macrophages, cells were cultured on TCP and treated with CMs supplemented with 10% FBS, 1% streptomycin‐amphotericin B‐penicillin. The medium was changed every second day.

### Cell Migration

Cell migration was evaluated by a scratch test. TSPCs were pre‐stained with Dil (Beyotime, C1036), seeded into 24‐well plates (5 × 10^4^ cells/well), and cultured until 90% confluence. A 1‐mL pipet tip was then used to make a scratch in the center of the cell monolayers. Floating cells were removed by rinsing in PBS. After that, cells were treated with CMs supplemented with 2.5% FBS and 1% streptomycin‐amphotericin B‐penicillin. The images were collected at 0, 12, and 24 h by a fluorescence microscope (Carl‐Zeiss, Germany). The healing area and the initial scratched wound area were quantified using ImageJ software and then the relative migration rates were determined.

### Live/Dead Staining

TSPCs (3 × 10^3^ cells/well) were seeded in a 96‐well plate (NEST, 701001) and cultured in RCM or ACM for 1 and 3 days. A calcein‐AM/PI double staining kit (Dojindo, Japan) was used to measure cell viability. TSPCs were incubated with the working solution at 37 °C with 5% CO_2_ for 20 min. Finally, cells were observed under a fluorescence microscope (Carl‐Zeiss, Germany) and representative pictures were taken. ImageJ was used to analyze cell viability.

### Cell Proliferation

TSPCs (3 × 10^3^ cells per well) were seeded in a 96‐well plate and cultured in CMs. Cell Counting Kit‐8 solution (CCK‐8, New Cell & Molecular Biotech, C6005) was incubated with cells for 1 h at corresponding time points. The produced formazan was measured using an 800 TS microplate reader (BioTek) at 450 nm.

### Inhibitors Treatment

TSPCs were seeded into 6‐ or 12‐well plates (NEST, 703001, 712001) and cultured in growth medium. When TSPCs reached to 90% confluence, the cells were treated with CMs or CMs combined with 20 µm U0126 (Selleck, S1102) to inhibit MAPK signaling pathway, or CMs combined with 40 µm RGD peptides (Selleck, S8008) to inhibit integrin signaling pathway. At the specified time points, the total RNA or protein was extracted for qPCR or western blot analysis respectively.

### RNA Extraction, Reverse Transcription, and qPCR

RNA was extracted using RNA prep Pure Cell/Bacteria Kit (Tiangen Biotech, DP430) and reversed transcription to cDNA by ReverTra Ace qPCR RT Master Mix (Toyobo, FSQ‐201). Subsequently, qPCR was performed according to the instructions provided by the manufacturer (SYBR Green Premix Pro Taq HS qPCR Kit, Solarbio Biotechnology (Hunan) Co., Ltd., AG11718). The sequences of used primers are summarized in **Table** **1**. Representative results are displayed as target gene expression normalized to the housekeeping gene.

**Table 1:** Primers used for qPCR.

Genes	5′‐3′	Primers
GAPDH (Glyceraldehyde‐3‐phosphate dehydrogenase)	Forward	GCAAGTTCAACGGCACAG
	Reverse	CGCCAGTAGACTCCACGAC
SCX (Scleraxis)	Forward	GCGAGAACACCCAGCCCAAAC
	Reverse	AAGCCATCACCCGCCTGTCCAT
TNMD (Tenomodulin)	Forward	GGGTGGTCCCGCAAGTGAAGGTG
	Reverse	GCCTCGACGACAGTAAATACAACAGT
MKX (Mohawk)	Forward	AAGTGAAATCTTGAGTCGAAAGG
	Reverse	GGGAGGACGTTATTGTTTGATG
PCNA (Proliferating cell nuclear antigen)	Forward	TAAGGGCTGAAGATAATGCTGAT
	Reverse	CCTGTTCTGGGATTCCAAGTT
ARG‐1 (Arginase‐1)	Forward	GTGAAGAACCCACGGTCTGT
	Reverse	CTGGTTGTCAGGGGAGTGTT
CD206 (Macrophage mannose receptor, MMR)	Forward	AGCTTCATCTTCGGGCCTTTG
	Reverse	GGTGACCACTCCTGCTGCTTTAG
IL‐10 (Interleukin 10)	Forward	CCAAGCCTTATCGGAAATGA
	Reverse	TTTTCACAGGGGAGAAATCG
IL‐1β (Interleukin‐1β)	Forward	AAGGAGAACCAAGCAACGACAAAA
	Reverse	TGGGGAACTCTGCAGACTCAAACT
CCR‐7 (C‐C Chemokine Receptor‐7)	Forward	ATGGACCCAGGTGTGCTTCT
	Reverse	TCAGTATCACCAGCCCGTTG
iNOS (Inducible nitric oxide synthase)	Forward	CCTGTGTTCCACCAGGAGAT
	Reverse	CCCTGGCTAGTGCTTCAGAC
ITGA1 (Integrin α1)	Forward	ACCAGTCAGCAGCTTCATTT
	Reverse	CACAGTTCCGTTCCAGTCATAG
ITGA11 (Integrin α11)	Forward	CATGGATGAGAGACGGTATATG
	Reverse	GTCCAGGACGTGGAAGTTGAT
ITGB1 (Integrin β1)	Forward	AAAATGGACGAAAGTGCTCTAAC
	Reverse	TGGGACTTGCTGGGATGC

### Immunofluorescence

TSPCs or RAW 264.7 (1 × 10^4^ cells per well) were seeded in a 96‐well plate and cultured in RCM or ACM for 2 days. Cells were fixed in 4% (w/v) paraformaldehyde (PFA) for 20 min, permeabilized with 0.1% (v/v) Triton X‐100 in PBS for 20 min, and blocked with 1% (w/v) BSA for 20 min. Subsequently, cells were incubated with rabbit anti‐Ki67 primary antibody (1:250, Proteintech, 27309‐1‐AP), anti‐SCX (1:500, Abcam, AB59655), anti‐TNMD (1:250, Abcam, AB203676), anti‐ARG‐1 (1:250, Proteintech, 16001‐1‐AP), anti‐CD206 (1:500, Proteintech, 18704‐1‐AP) and anti‐iNOS (1:300, Affinity Biosciences, AF0199) at 4 °C overnight. On the second day, cells were washed and incubated with 488‐conjugated goat anti‐rabbit IgG (Proteintech, SA00013‐2) for 1 h at room temperature. The nuclei of the cells were revealed by 4′,6‐diamidino‐2‐phenylindole (DAPI; Beyotime, C1002). The number of Ki67+ cells was presented as a percentage of the total cell number. ImageJ was used to quantify the SCX, TNMD, ARG‐1, CD206, iNOS results based on the average intensity and positive area of representative images. “3D surface plot” plugins, from ImageJ, were used to create the 3D heatmap images of the fluorescence result.

### Western Blot Assay

Collected cell samples were lysed at 4 °C in RIPA lysis buffer (Beyotime, P013B) mixed with Phenylmethanesulfonyl fluoride (PMSF, Beyotime, ST505) and Protease and phosphatase inhibitor cocktail (Beyotime, P1045). After the total protein was extracted, the concentration was quantified using the Enhanced BCA Protein Assay Kit (Beyotime, P0010). For western blot analysis, 15 µg protein was loaded for each sample, separated by 10% SDS‐PAGE, and transferred to PVDF membranes. After the membranes were blocked in QuickBlock Blocking Buffer (Beyotime, P0252) for 15 min, the primary antibodies against p‐MEK (1:1000, CST, 9154), MEK (1:1000, CST, 8727), p‐ERK (1:2000, CST, 4370), ERK (1:1000, CST, 4695), β‐arrestin (1:1000, CST, 30036), and GAPDH (1:200000, Proteintech, 60004‐1‐lg) were used to incubate the membranes. After washing, the membranes were incubated with a horseradish peroxidase‐labeled secondary antibody (Beyotime, A0208; Proteintech, SA00001‐1) for 1 h at room temperature. Ultimately, protein bands were visualized after incubation with a chemiluminescence kit (Vazyme, E422‐01). The expression levels were quantified by analyzing the mean gray value (IntDen/Area) of the bands using ImageJ software.

### Animal Experiment

Sixteen adult female Sprague‐Dawley rats weighing 200–220 grams aged 6–8 weeks were used in this study, in which eight for the following histological examination, transmission electron microscopy (TEM), and immunohistochemical staining, and the other eight for gait analysis and mechanical stretch test. Southeast University's animal experimentation ethics committee approved the study protocol (permit No.20220530060). All experiments were designed in accordance with the Animal Research: Reporting of In Vivo Experiments (ARRIVE) guidelines. All rats were maintained on a normal 12 h light /12 h dark day/night light cycle with free access to food and water. Under general anesthesia, patellar tendon defects of 6 mm in length and 1 mm in width were created.^[^ ^74^ ^]^ The collected CMs were lyophilized and resuspended with 10% SilMA (EFL‐SilMA‐001). Subsequently, 30 µL liquid of CMs‐SilMA was carefully pipetted onto the window defect area of patellar tendon. Rats were randomly selected to receive RCMs‐SilMA solution in one leg and ACMs‐SilMA solution in the other. The solutions were in situ photocrosslinked into hydrogel state by using an ultraviolet (UV) irradiation (wavelength: 365–370 nm; light strength: 50 mW cm^−2^) for 30 s. After gelation, the formed hydrogel was fixed in the defect area and unlikely to shift. After 1 or 4 weeks, rats were sacrificed by intraperitoneal injection of an overdose of 1% pentobarbital sodium (150 mg kg^−1^). The rats were confirmed dead by observing cardiac arrest for 10 min and specimens were harvested for the following evaluation.

### Histological Examination

Collected specimens were fixed in 10% (v/v) neutral buffered formalin, dehydrated through an alcohol gradient, embedded in paraffin, and sectioned by a microtome. Hematoxylin and eosin (H&E) staining and Masson trichrome staining were carried out according to standard procedures to examine the general appearance of repaired tissues. To quantify the repair effect of injured tendons, histological scoring was done blinded based on six parameters of H&E staining pictures, which include fiber structure, fiber arrangement, nuclear roundness, vascular numbers, inflammatory cell infiltration, and cell quantity.^[^ ^7^ ^]^

### Picrosirius Red Staining

3 µm serial slices were stained with Sirius Red Stain Kit (Solarbio, G1472) for 30 min. The stained samples were observed and imaged by using a polarized light microscope (Olympus) to identify collagen I (red or light orange) and collagen III fibers (green). For quantitative analysis, the area of each color was measured by ImageJ software.^[^ ^75^ ^]^

### Transmission Electron Microscopy (TEM)

For TEM imaging to assess the collagen fibrils’ diameter, tissue specimens were fixed and processed by standard procedures. Briefly, specimens were fixed in 2.5% glutaraldehyde for 24 h, post‐fixed in 1% osmium tetroxide, dehydrated through a graded series of alcohols, and then embedded in Araldite resin. After ultramicrotomy, the ultrathin sections were stained with uranyl acetate and lead citrate, and examined under a TEM. The diameters of 100 collagen fibrils in each group were measured by ImageJ software.

### Immunohistochemistry

Immunohistochemical staining was performed to analyze protein expression levels. Sections were deparaffinized and hydrated through a graded series of alcohol. The antigen thermal repair was performed with Tris‐EDTA at 60 °C for 6 h and then incubated in 3% hydrogen peroxide at room temperature for 20 min. After blocked, the slices were incubated with primary rabbit polyclonal antibodies at 4 °C overnight and then incubated with a horseradish peroxidase‐labeled secondary antibody at 37 °C for 30 min. Next, 3,3‐diaminobenzidine (DAB) was added at room temperature for 30 min, and the slices were then stained with hematoxylin for 15 s. Finally, the slices were washed and dehydrated in gradient alcohol solutions, mounted with neutral balsam, and observed using an optical microscope. The primary antibodies used in this study were rabbit anti‐SCX (Abcam, AB59655), rabbit anti‐TNMD (Abcam, AB203676), rabbit anti‐collagen type I (COL I) (Proteintech, 14695‐1‐AP), rabbit anti‐collagen type III (COL III) (Proteintech, 22734‐1‐AP), rabbit anti‐ARG‐1 (Proteintech, 16001‐1‐AP), rabbit anti‐CD206 (Proteintech, 18704‐1‐AP), rabbit anti‐iNOS (Affinity Biosciences, AF0199). To quantify the results of immunohistochemistry, six representative images each group were analyzed by ImageJ. The positive area was measured by using the plugin IHC Toolbox in ImageJ software to split the actual positive region. The total area was also measured by ImageJ. The ratio was calculated by positive area/total area.

### Gait Analysis

Eight rats were selected randomly to perform gait experiment at 2 weeks and 4 weeks after surgery by using the CatWalk System (CatWalk XT, Noldus Information Technology, Netherlands). The rat was placed individually in the walkway and walked freely from one side to the other side while the gait changes were recorded and analyzed by the software. The left and right hind limbs of experimental rats were examined. The average area (average area of a paw contacting the glass), stand time (average seconds of a paw contacting the glass), mean intensity (average pressure of a paw contacting the glass), swing speed (average speed of the paw swings away from the contact surface), stand/swing time ratio, and stand/step cycle time (%) were recorded and compared between groups.

### Biomechanical Evaluation

Biomechanical evaluation was performed at 4 weeks post‐surgery. After rats were sacrificed, ≈1 cm of peritendinous tissue above and below the surgical site of the patellar tendon was excised. The biological strength of the repaired tendon was evaluated with a longitudinal load by an electric universal testing machine (UTM2502 with a 50 N sensor; Sunstest, Shenzhen, China). The tissues were stretched at a strain rate of 5 mm min^−1^. With stress‐strain curves generated by software, the elastic modulus was calculated according to the slopes of the linear regions. The max force and ultimate tensile strength were recorded at the point of rupture.

### Proteomic Analysis

Proteomic analysis based on the inbuilt label‐free quantification (LFQ) was conducted to examine the secretome of ADSCs cultured on randomly oriented or aligned silk scaffolds for 24 h. The proteomic data were analyzed on the DAVID website (https://david.ncifcrf.gov/) and OmicStudio tools (https://www.omicstudio.cn/). For hierarchical cluster analysis and heatmap generation, protein expression values were z‐score normalized across samples. The differentially expressed proteins (DEPs) with a *p*‐value less than 0.05 were recognized and used for GO and KEGG enrichment analysis. The mass spectrometry proteomics data have been deposited to the ProteomeXchange Consortium (http://proteomecentral.proteomexchange.org) via the iProX partner repository with the dataset identifier PXD047809.

### Statistical Analysis

Data were presented as mean ± SD unless specially declared. The statistical difference between the two groups was determined using Student's *t*‐tests, and one‐way analysis of variance (ANOVA) with Tukey's multiple comparisons was applied to the comparison of more than two groups in GraphPad Prism 9. In animal experiments, one leg was randomly selected as the experimental group (ACM) and the other leg as the control group (RCM). Therefore, paired *t*‐test was used for gait analysis and tendon mechanical test. Representative results were shown for all experiments. Statistical significance was defined as a *p*‐value of less than 0.05 for all comparisons.

## Conflict of Interest

The authors declare no conflict of interest.

## Author Contributions

J.C., Q.L., J.H., and W.Z. conceived the idea and designed the experiments. J.C., Q.L., L.B., and W.Z. wrote the manuscript. J.C., Q.L., C.L., H.L., Y.L., M.W., H.Z., J.Z., L.H., Y.S., Z.C., and Q.M. performed experiments. J.C., Q.L., J.H., C.L., M.W., H.Z., Q.M., and W.Z. analyzed the data. JC and WZ financially supported the study. All authors read and approved the final manuscript.

## Ethics Approval and Consent to Participate

The study protocol of animal experiments was approved by the animal experimental ethics committee of Southeast University (permit No. 20220530060).

## Supporting information



Supporting Information

## Data Availability

The data that support the findings of this study are available in the supplementary material of this article.
