# Supplementary material for: Enhancing Tendon Regeneration: Investigating the Impact of Topography on the Secretome of Adipose‐Derived Stem Cells
Source: Adv Sci (Weinh). 2025 Mar 17;12(18):2417447. doi: 10.1002/advs.202417447 (PMC12079404; doi:10.1002/advs.202417447)
Supplement: Supplementary file 1 — Supporting Information [file ADVS-12-2417447-s001.docx]

Supporting Information

**Enhancing Tendon Regeneration: Investigating the Impact of Topography on the Secretome of Adipose-Derived Stem Cells**

Qiuzi Long, Chuanquan Liu, Haotian Zheng, Mingyue Wang, Hanmei Liu, Yue Liu, Zhicheng Cao, Yuzhi Sun, Qingyun Mo, Ludvig J. Backman, Jialin Zhu, Lizhi Hu, Jinlong Huang*, Wei Zhang*, Jialin Chen*


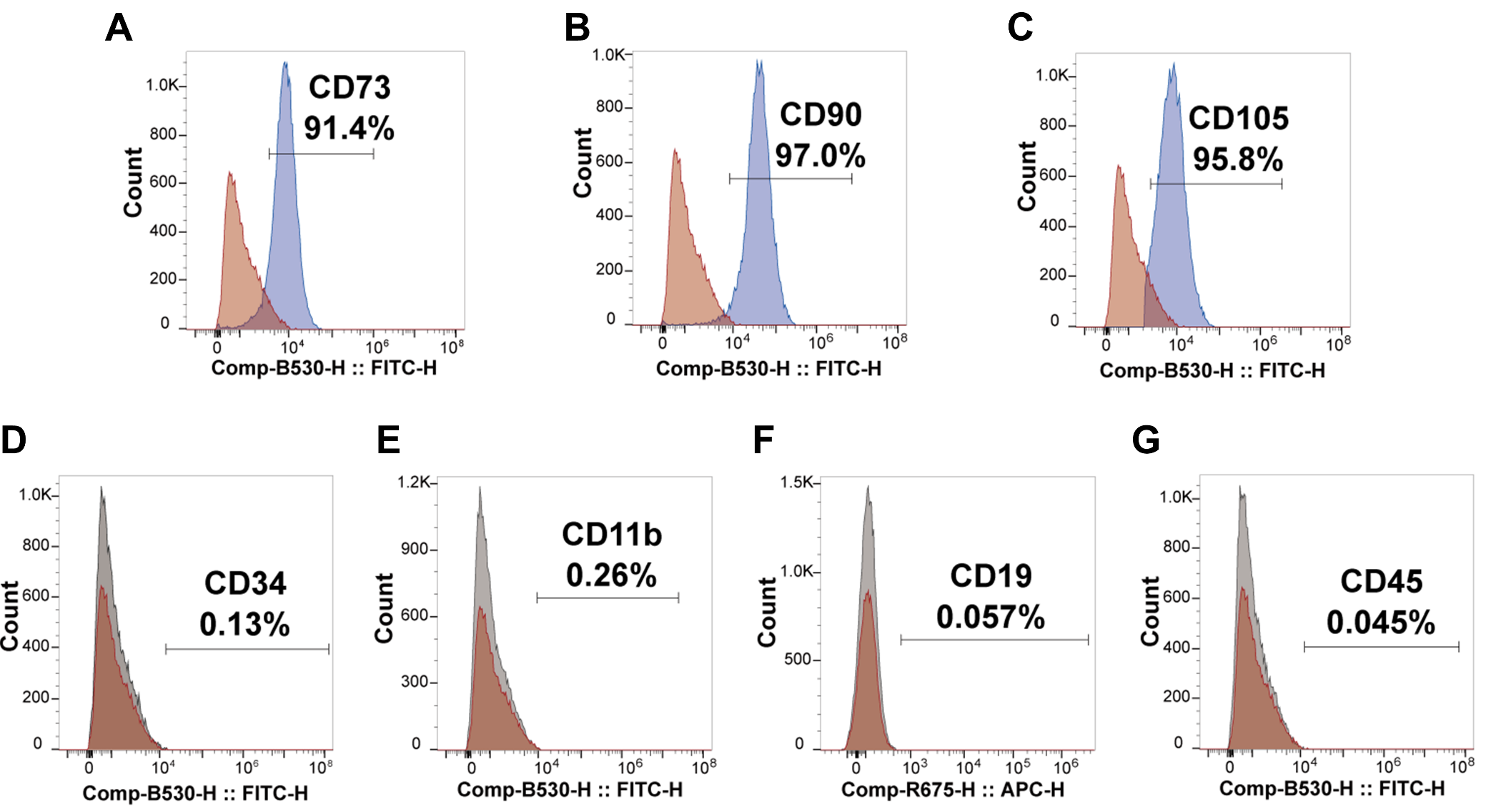


**Figure S1.** Flow cytometric analysis of the expression of MSCs surface markers in ADSCs. (A): CD73. (B): CD90. (C): CD105. (D): CD34. (E): CD11b. (F): CD19. (G): CD45.


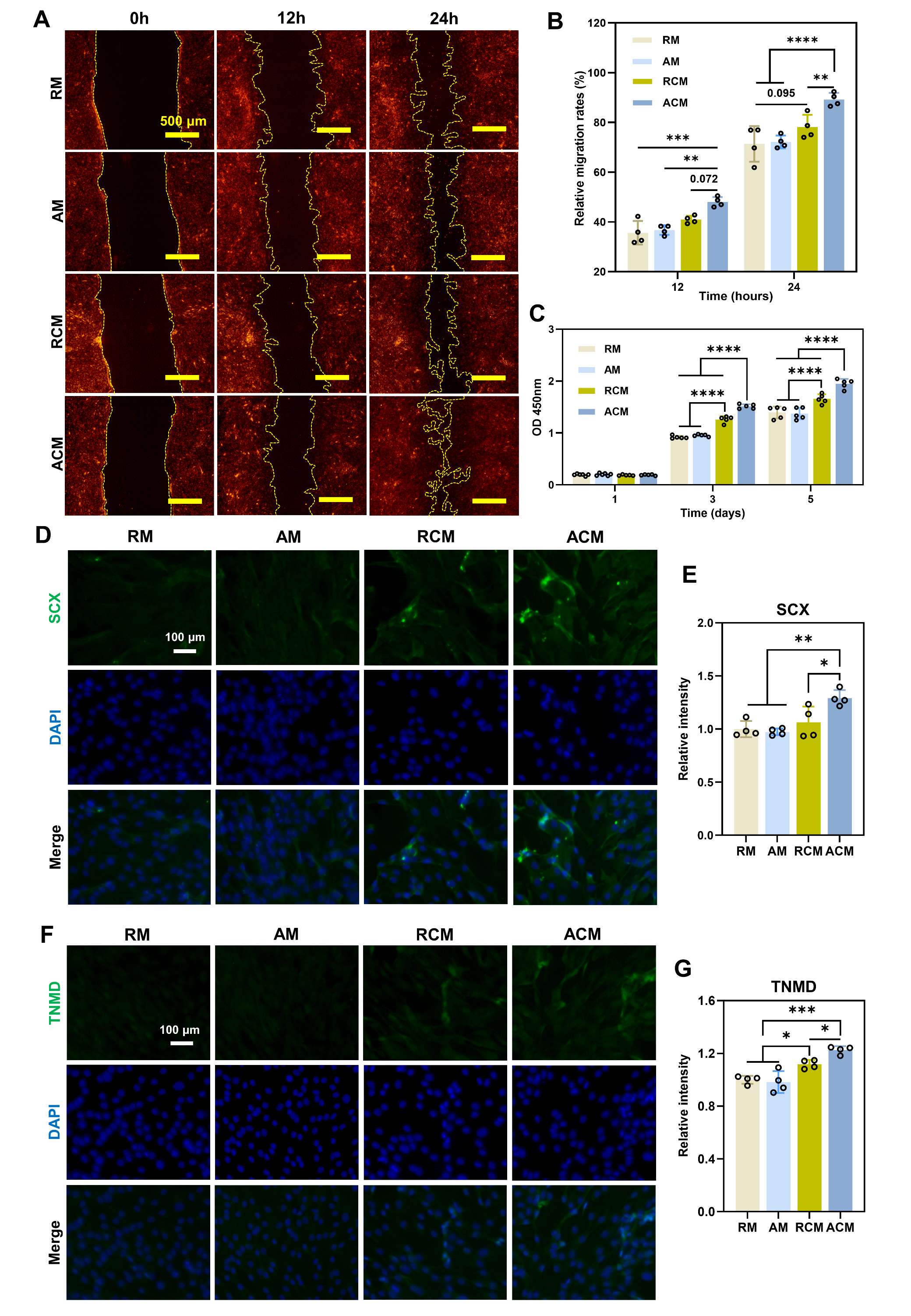
 **Figure S2.** Effect of CMs on TSPCs cell migration, proliferation and tendinous differentiation. The TSPCs were cultured in RM, AM, RCM and ACM. (A): Cell migration of TSPCs treated with RM, AM, RCM or ACM using scratch assay at 0, 12, and 24 h. TSPCs were pre-stained using Dil. Scale bars = 500 μm. (B): The relative migration rate was quantified and compared, n = 4 technically independent samples for each group, **p < 0.01, ***p < 0.001, ****p < 0.0001. (C): Proliferation of TSPCs in RM, AM, RCM and ACM for 1, 3, and 5 days measured by CCK-8, n = 5 technically independent samples for each group, ****p < 0.0001. (D, F): The expression of SCX and TNMD were evaluated by immunofluorescence staining. Scale bars = 100 μm. (E, G): Relative intensity of SCX and TNMD were quantified, n = 4 randomly-selected microscopic images per group, *p < 0.05, **p < 0.01, ***p < 0.001.


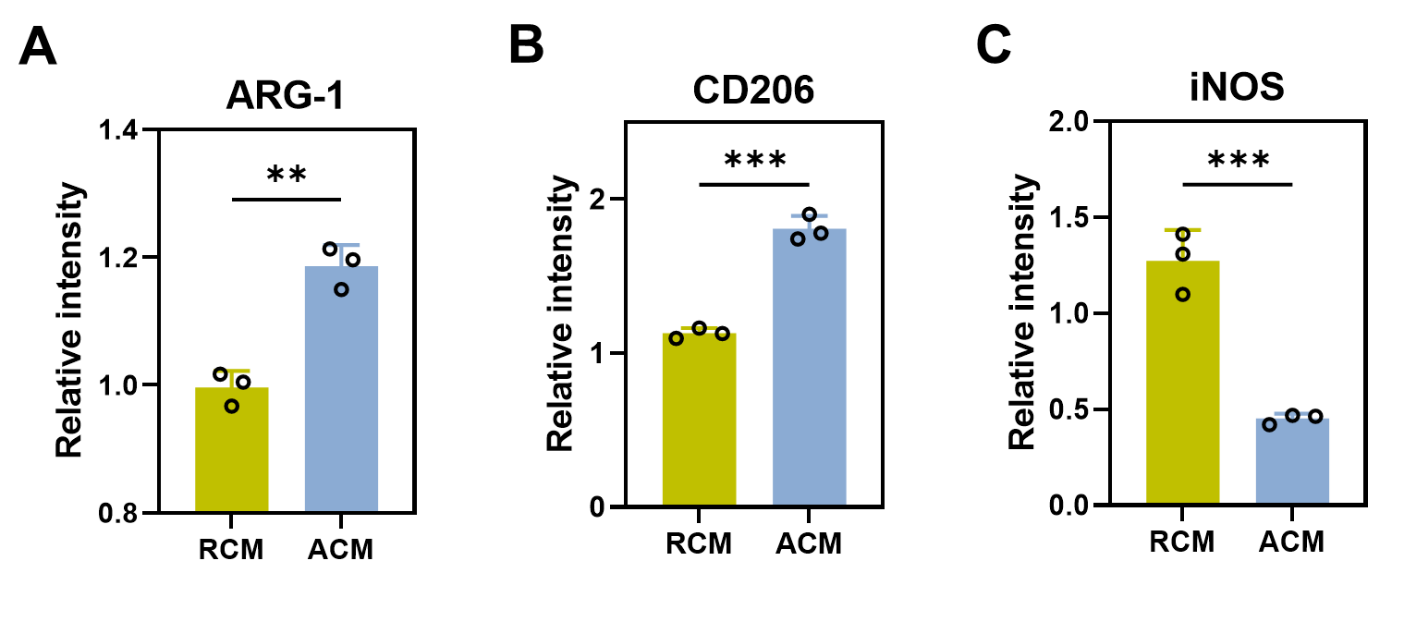


**Figure S3.** The fluorescence intensity quantification of immunofluorescence staining result in ARG-1, CD206, and iNOS by ImageJ. (A-C): Relative intensity of ARG-1, CD206, and iNOS were quantified, n = 3 randomly-selected microscopic images per group, **p < 0.01, ***p < 0.001.


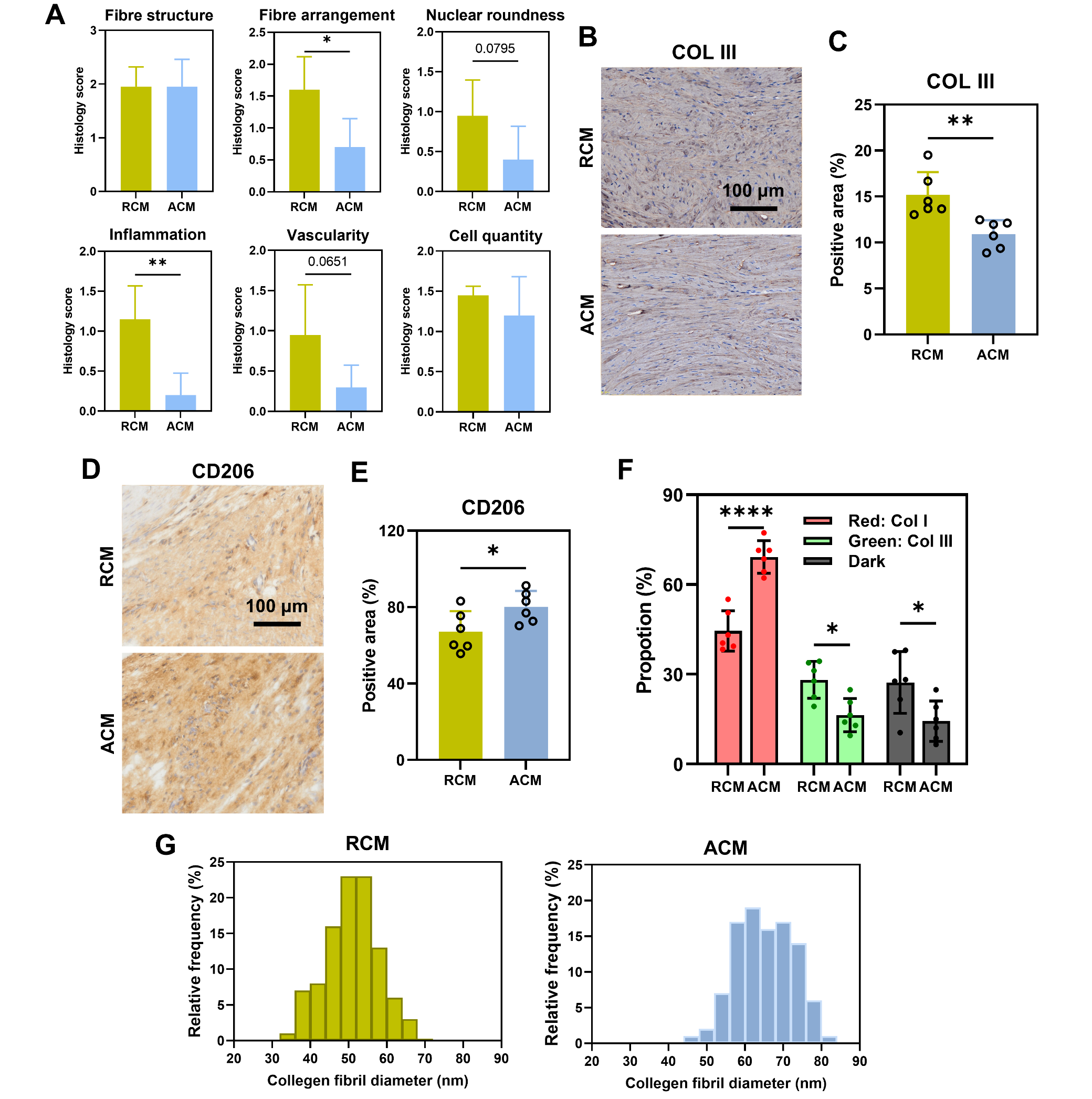


**Figure S4.** (A): Histological scoring was done based on six parameters of H&E staining pictures, which includes fiber structure, fiber arrangement, nuclear roundness, inflammation, vascularity, and cell quantity, *p < 0.05, **p < 0.01. (B): Immunohistochemical staining of COL III. Scale bar = 100 μm. (C): The percentage of COL III immunohistochemical positive area in the pictures. n = 6 randomly-selected microscopic images per group. (D): The immunohistochemical staining of the macrophage polarization marker CD206 (M2, anti-inflammatory) in repaired tendons. Scale bar = 100 μm. (E): The percentage of CD206 immunohistochemical positive area in the pictures, *p < 0.05. n = 6 randomly-selected microscopic images per group. (F): The proportion rates of collagen I (red or light orange area), collagen III fibrils (green area), and dark area were quantified and compared. n = 6 randomly-selected microscopic images per group, *p < 0.05, ****p < 0.0001. (G): The histogram of collagen fibrils’ diameters in RCM and ACM group.


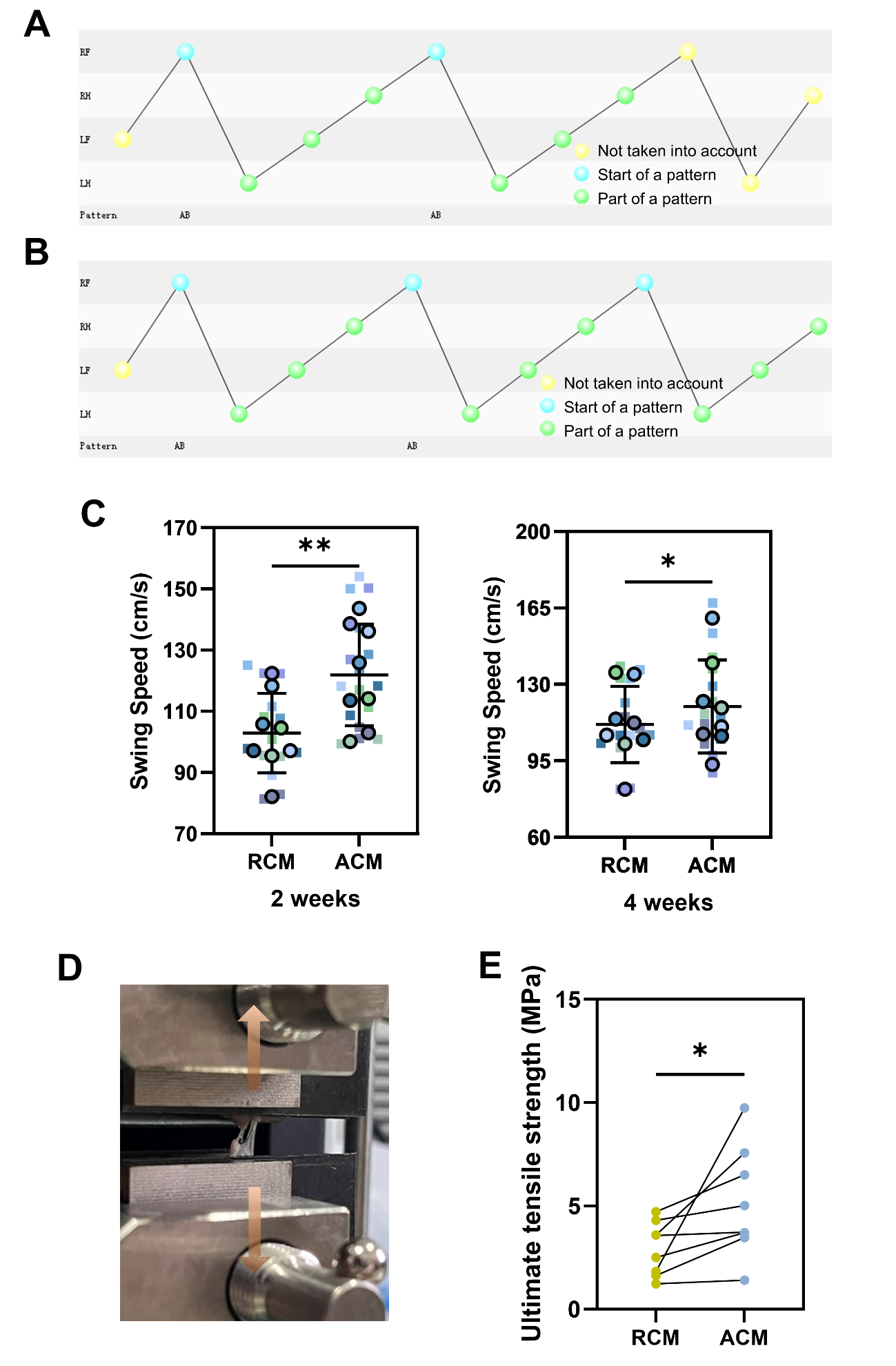


**Figure S5.** The representative footfall pattern in CatWalk at two weeks (A) and four weeks (B). (C): Quantification of swing speed at 2 weeks and 4 weeks. n = 16 randomly-selected results from 8 biological samples, *p < 0.05, **p < 0.01. (D): The photograph of mechanic test. (E): Comparison of ultimate tensile strength between groups. n = 8 biologicaly independent samples, *p < 0.05.


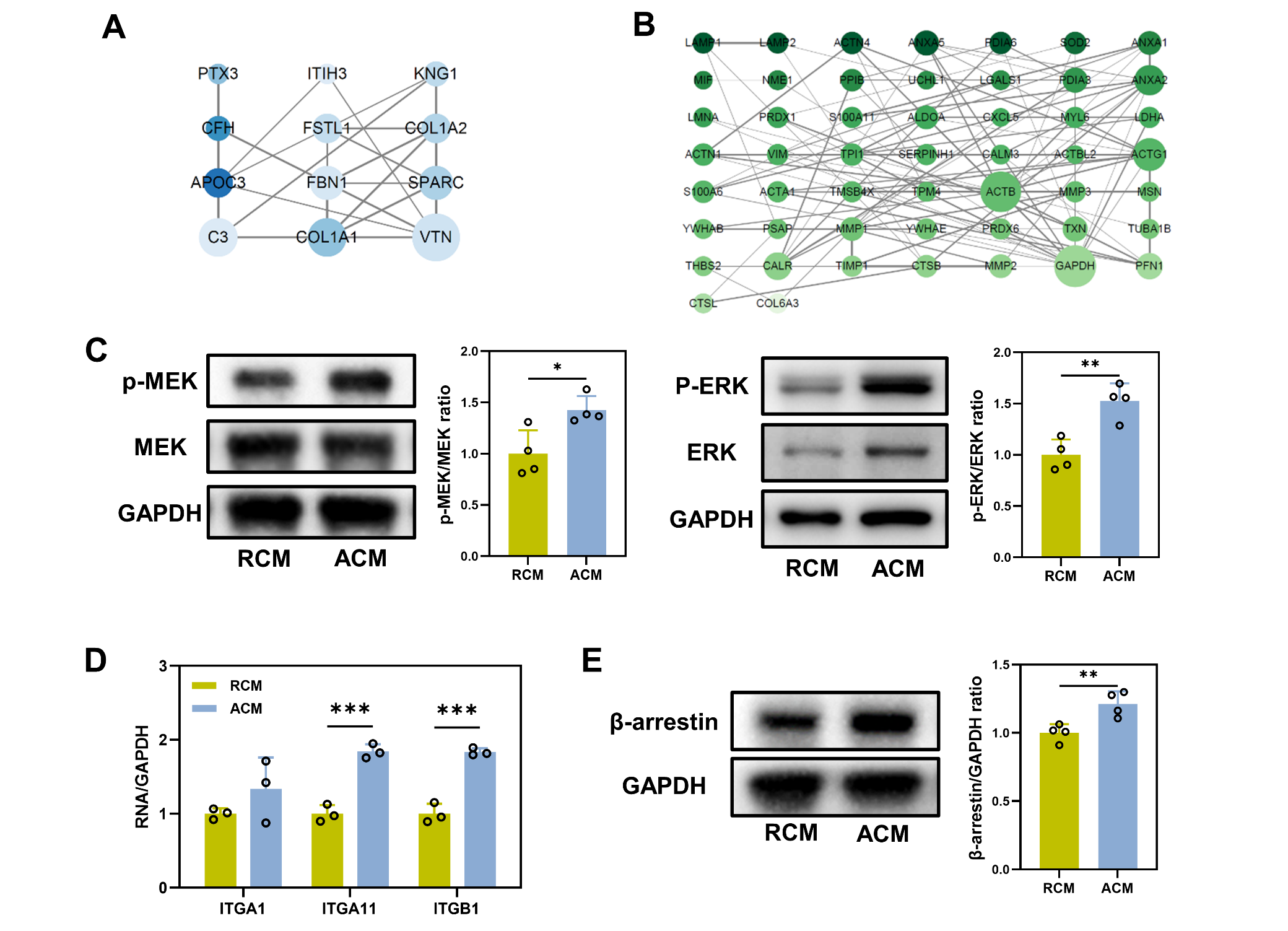


**Figure S6.** (A and B): The protein interaction networks of DEPs. A larger size of protein node represents a higher frequency in the protein networks. A deeper color of nodes represents a higher or lower fold change of DEPs. The upregulated proteins in the ACM group are presented in the blue circle while the downregulated proteins are in the green circle. (C): Western blot and quantitative analysis of p-MEK, MEK, p-ERK and ERK in TSPCs after RCM and ACM treatment for 18 hours. n = 4 technically independent samples for each group, *p < 0.05, **p < 0.01. (D): The mRNA levels of integrin-related genes of TSPCs on day 2 using qPCR. n = 3 technically independent samples for each group, ***p < 0.001. (E): Western blot and quantitative analysis of β-arrestin in TSPCs after the treatment of RCM and ACM for 12 hours. n = 4 technically independent samples for each group, **p < 0.01.
